# Identifying a panel of genes/proteins/miRNAs modulated by arsenicals in bladder, prostate, kidney cancers

**DOI:** 10.1038/s41598-018-28739-6

**Published:** 2018-07-10

**Authors:** Andrea Polo, Silvia Marchese, Giuseppina De Petro, Maurizio Montella, Gennaro Ciliberto, Alfredo Budillon, Susan Costantini

**Affiliations:** 1 Experimental Pharmacology Unit, Istituto Nazionale Tumori - IRCCS - Fondazione G. Pascale, Napoli, Italy; 2 Dipartimento di Medicina Molecolare e Traslazionale, Università di Brescia, Brescia, Italy; 3 Epidemiology Unit, Istituto Nazionale Tumori - IRCCS - Fondazione G. Pascale, Napoli, Italy; 4 Scientific Directorate, IRCCS Istituto Nazionale Tumori “Regina Elena”, Roma, Italy

## Abstract

Arsenic and arsenic-derivative compounds, named as arsenicals, represent a worldwide problem for their effect on the human health and, in particular, for their capability to increase the risk of developing cancer such as kidney, bladder and prostate cancer. The main source of arsenical exposure is drinking water. Nowadays, it is well known that the chronic exposure to arsenicals leads to a series of epigenetic alterations that have a role in arsenic-induced effects on human health including cancer. Based on these observations, the aim of our study was to select by network analysis the genes/proteins/miRNAs implicated in kidney, bladder and prostate cancer development upon arsenical exposure. From this analysis we identified: (i) the nodes linking the three molecular networks specific for kidney, bladder and prostate cancer; (ii) the relative HUB nodes (RXRA, MAP3K7, NR3C1, PABPC1, NDRG1, RELA and CTNNB1) that link the three cancer networks; (iii) the miRNAs able to target these HUB nodes. In conclusion, we highlighted a panel of potential molecules related to the molecular mechanisms of arsenical-induced cancerogenesis and suggest their utility as biomarkers or therapeutic targets.

## Introduction

Arsenic is a commonly occurring metalloid, found in nature mainly in its inorganic form. Inorganic arsenicals are found in soils, from which in certain condition they can be transferred to groundwater, and consequently in agricultural product, like rice and vegetable. In addition to these natural sources, anthropogenic release of arsenic is common in association with different kind of industries, like mines and electronic industries. Therefore, assumption of arsenicals causes a wide range of adverse effects on humans and animals, ranging from dermal lesions, cardiovascular diseases, diabetes, neuropathy and cancer^1^. In particular, carcinogenic potential of arsenicals was reported for the first time already at the end of 19th century, when medical arsenic showed to cause skin cancer. Since then, arsenic was recognized as a multisite and multifactorial carcinogen by International Agency for Research on Cancer (IARC) and National Toxicology Program (NTP), targeting several organs mainly lung and bladder^2^.

However, the major source of arsenicals exposure is the drinking water which has the higher level of arsenic rather than in food, atmosphere and industrial sources^2,3^. Many epidemiologic studies evidenced its involvement in bladder cancer (BlC) and also in prostate (PrC) and kidney (KiC) cancers^2,3^. For these reasons we focused on these three cancers. While data on kidney and bladder cancer are fairly indicative of the effect of arsenic, the data on the prostate are less clear. However, already in 1999, PrC incidence resulted to be increased in 22 areas of Australia with elevated arsenic exposure^4^. In 2004 an increase of mortality for PrC was reported in the high arsenic-contaminated areas of West Taiwan^2,3^. More recently, higher PrC risk with a linear correlation between drinking water dose and response was found in the areas of Illinois State in USA^5^. Some “*in vitro*” studies on human prostate epithelial cells (RWPE-1) demonstrated the carcinogenic transformation of these cells after few months of exposure to sodium arsenite^6^ and to inorganic arsenite that resulted able to induce also over-expression of metalloproteinase-9 (MMP9)^7^. In 2014, some Authors showed that methylarsonous acid and inorganic arsenic caused an acquired malignant phenotype in methylation-deficient cells and that inorganic arsenic likely has both genotoxic and non-genotoxic mechanisms dictated by the ability of target cells to methylate arsenic^8^. Moreover, a possible synergy between arsenic and estrogen-like compounds released by plastic to develop PrC has been reported because arsenic can interact with estrogen receptors and activate estrogen-regulated genes^9^.

As pointed out above, several studies found a correlation between high incidence of KiC and arsenic exposure in areas with higher levels of arsenic contamination^10^. However, ecological studies demonstrated a relationship between arsenic and mortality in KiC in 42 villages of Southwest Taiwan^11^, and 26 counties of Cordoba in Argentina that have higher arsenic contamination areas^12^. Moreover, studies conducted on urine samples of the population reside in Taipei City and on histological and cytological sample of renal biopsies in Bangladesh showed that high levels of arsenic increased the development risk of KiC^13,14^. Experimental studies in rat kidney demonstrated that the chronic exposure to low level of arsenic induced an increased secretion of MMP2 and MMP9 leading to a more rapid proliferation of KiC that tend to acquire a cancerogenic phenotype^15^.

Moreover, epidemiological studies conducted in South American populations evidenced elevated risk for BlC in relation with the high concentration of arsenic^16^. A case study, conducted in Argentina and Chile on patients exposed to arsenic compounds, found chromosomal alterations in DNA of BlC patients associated with arsenic exposure and suggested that these alterations are also associated with tumor stage and grade^17^. The evaluation of the levels of arsenic and its compounds in the urine of BlC patients living in Taiwan, demonstrated an higher BlC risk upon arsenic exposure^18^.

Although, more experiment are necessary to elucidate the molecular mechanism of action of the arsenic and its derivative compounds, all these data suggest the implication of arsenic in the development and initiation of BlC, KiC and PrC.

In this context it is important to underline that interaction network analysis applied to data related to gene, protein and miRNA expression is playing an increasing and important role in the understanding of physiological processes leading to the cancer initiation and progression^19^. From this analysis, genes, proteins and miRNAs can be described by nodes and the interactions between them by edges. Considering centrality and topology measures on the networks, it is possible to identify HUB nodes, which are the nodes with the strongest coordination role, that can represent key molecules in cancer development and thus putative diagnostic/prognostic markers or therapeutic targets^19^.

Hence, the aim of our work was to select the genes/proteins/miRNAs modulated by arsenicals in BlC, PrC and KiC. Firstly, comparative toxicogenomics database (CTD) was used to extract the list of the genes/proteins modulated by the arsenicals in KiC and PrC cancers. On the basis of these data we created the related networks and compared them with the BlC network, recently published by our group that resulted to comprise nine HUB nodes (PSMB2, TNF, FANCA, KRAS, CCNE1, PABPC1, BIRC3, ERCC4 and PRSS3) (Table 1)^20^. Then, we merged the three networks and analyzed in more detail the sub-network that links PrC, BlC and KiC networks. In this way, we identified the related HUB nodes and the miRNAs able to target these HUB nodes.

**Table 1:** Mutational status of HUB nodes in the networks obtained for proteins modulated by arsenicals in Bladder, Kidney and Prostate cancer.

HUB	BlC	HUB	KiC	HUB	PrC
PSMB2	1.2%	BAP1	17%	RXRA	1.6%
TNF	2.7%	RELA	0.9%	VEGFA	1%
FANCA	4%	TP53	3%	NR3C1	1.2%
KRAS	5%	BIRC5	0.6%	PDE4D	8%
CCNE1	8%	IL4R	0.6%	MAP3K7	15%
PABPC1	17%	VHL	46%	TP53	18%
BIRC3	5%	MLLT10	1.5%	PHGDH	1%
ERCC4	4%	PIDD1	0%	NDRG1	7%
PRSS3	1.5%				

## Results

### Selection of arsenicals implicated in KiC and PrC and their related modulated proteins

We used the Comparative Toxicogenomics Database (CTD) in order to extract firstly the list of arsenicals implicated in KiC and PrC and, then, the list of proteins modulated by these compounds in these cancers to build the relative interaction networks. CTD analysis has evidenced the presence of two hundred and eighteen arsenicals belonging to arsenical family. Among these compounds, only for forty-four arsenicals the proteins modulated by them are reported. Moreover, only for eighteen arsenicals there are information about proteins modulated by them in KiC and PrC. Considering that we published recently an interaction network analysis on the proteins modulated by arsenicals in BlC^20^, we decided to select, among these eighteen arsenicals, the seven compounds (at exception of arsenotriglutathione for which there are not proteins modulated in PrC and KiC) that were object of that paper because our aim was to compare the interaction networks obtained for KiC, PrC and BlC (Supplementary Table 1).

For each cancer, we extracted the list of proteins modulated by each compound. Since there were differences between the various arsenic compounds and the modulated proteins, we used for the network analysis the total list of the proteins modulated by seven arsenicals in each cancer (Supplementary Table 1).

### Analysis of arsenicals-proteins interaction network in Kidney Cancer

The list of ninety-six proteins modulated by the arsenicals (Supplementary Table 1) and implicated in KiC was extracted (Supplementary Table 2). To demonstrate that these proteins and their related genes have been already found to be implicated in KiC, we performed a meta-analysis on gene expression data by cbioPortal tool using TCGA_KIRC dataset including 538 patient cases. This analysis showed that: (i) 24.35% of cases presents mutations in the genes related to the ninety-six proteins; (ii) 2.97% of cases presents amplifications of these genes; (iii) 2.6% of cases presents deep deletions; and (iv) 45.17% of cases showed multiple alterations (Supplementary Fig. S1). Therefore, it confirmed the involvement of these genes/proteins in KiC.

In parallel, a functional analysis showed that these proteins are involved in some important metabolic pathways such as AMPK, ErbB, HIF-1, Insulin, mTOR, MAPK, NF-kappa B, PI3K-Akt, p53, TNF, Ras, and VEGF signaling, Apoptosis and Cytokine-cytokine receptor interaction (Supplementary Table 3).

In order to identify the proteins playing the most important role in KiC, we mapped the ninety-six proteins on the complete human interactome^21^ and obtained a network composed of 6108 nodes (proteins) and 22358 edges (interactions). A detailed analysis of the statistical centrality and topological measures on the network has permitted to evidence its effectiveness and its robustness (Table 2)^22–26^. In detail, the obtained network is centralized with a value of 0.119, where higher the value of centralization much more the network is concentrated in the center with an overall integration towards the high degree nodes^22–26^. The network density, which describes the portion of the potential connections in a network that are actual connections, as a measure of network effectiveness^22–26^, has value of 0.001 whereas its tendency to contain HUB nodes is shown by the high value of the heterogeneity equal to 4.778 and average number of neighbors equal to 4.051. The characteristic path length is 3.875 indicating that our network follows the small-world rule because the characteristic path length is very short^22^. As shown in Supplementary Fig. 2, the plot of the node degree distribution showed a decreasing trend demonstrating that our network has scale free property indicating that “riches get richer”^20,22–26^. Also the clustering coefficient graph showed a decreasing trend with a clustering coefficient equal to 0.097 and reflects the tendency of a network to contain HUB nodes^20,22–26^. This finding is also confirmed by the plot related to stress centrality where a great number of nodes is traversed by a high number of shortest paths and by the closeness centrality that has an increasing trend. Moreover, we evaluated the betweenness centrality that provides inferences on the importance of proteins on the basis of load placed on the given node in the network, and, hence, information about the core skeleton of the network. Betweenness centrality demonstrated an increasing trend confirming the presence of HUB nodes.

**Table 2:** Detailed analysis of the statistical centrality and topological measures on the networks.

Statistical analysis	KiC	PrC	linking sub-network between KiC, PrC and BlC
Clustering coefficient	0.097	0.088	0.022
Network centralization	0.119	0.079	0.217
Characteristic path length	3.875	3.859	4.091
Avg. number of neighbors	4.051	5.120	2.291
Network density	0.001	0.001	0.002
Network heterogeneity	4.778	4.109	4.638

On the basis of the analysis of the statistical centrality and topological measures on the network, the following eight HUB nodes were identified: BAP1, RELA, TP53, BIRC5, VHL, IL4R, MLLT10 and PIDD1. Then, we extracted the interactions between HUB nodes and obtained a sub-network composed of 576 nodes and 609 interactions that has been then merged with the related HUB-HUB interaction sub-networks specific for BlC and PrC in order to find the nodes linking KiC, BlC and PrC.

Moreover, to verify that ninety-six proteins modulated by the arsenicals in KiC are really correlated between them and the obtained network by Cytoscape was based on physical interactions and/or regulatory effects, experimentally proven, we performed a protein-protein network analysis also by STRING tool (https://string-db.org/). STRING network showed that about 14.6% (corresponding to fourteen proteins) and 26.9% (corresponding to twenty-five proteins) out the ninety-six proteins had no relation with confidence values of 0.4 and 0.7, respectively. Certainly, these results confirmed that the ninety-six proteins modulated by the arsenicals in KiC are correlated between them as suggested also by the significant p-values in the pathway analysis (Supplementary Fig. 3 and Supplementary Table 3).

Taking advantage of the TCGA_KIRC dataset we evidenced that seven out of the eight HUB nodes (BAP1, BIRC5, IL4R, MLLT10, RELA, TP53 and VHL) are mutated in KiC with frequency ranging from 0.6% to 46% (Table 1). The two most altered nodes are VHL and BAP1 with mutation frequencies equal to 46% and 17%, respectively, suggesting the involvement of these nodes in KiC cancerogenesis.

### Analysis of arsenicals-proteins interaction network in Prostate Cancer

About PrC we selected three hundred and thirteen proteins modulated by the arsenicals (Supplementary Table 4). A meta-analysis performed on gene expression data by cbioPortal tool using TCGA_PRAD dataset including 501 patient cases has showed that: (i) 0.6% of cases presents only mutations in the genes related to the three hundred and thirteen proteins; (ii) 0.2% of cases presents only amplifications of these genes; (iii) 0.4% of cases presents only deep deletions; and (iv) 89.42% of cases showed multiple alterations (Supplementary Fig. 1). Also in this case, this analysis confirmed the involvement of these genes/proteins in PrC. These proteins resulted to be involved in some important pathways among which FoxO, PI3K-Akt, HIF-1, p53 signaling pathways, Apoptosis, Cytokine-cytokine receptor interaction, and Glutathione metabolism (Supplementary Table 5).

We applied the same procedure described for KiC to map the identified proteins on the complete human interactome^21^ and to extrapolate their molecular network interaction to identify HUB nodes. The obtained network was composed of 9782 nodes (proteins) and 44258 edges (interactions). It has a centralization value of 0.079, a network density of 0.001, a heterogeneity value of 5.171, a characteristic path length of 3.859, a clustering coefficient of 0.088 and an average number of neighbors of 5.120 (Table 2). Overall these data evidenced that the network effectiveness is elevated with nodes that are highly correlated between them, and the network is of small world type characterized by short path lengths.

Moreover, the plots of the node degree distribution, clustering coefficient, stress centrality, closeness centrality and betweenness centrality^22–26^ (Supplementary Fig. 4) confirmed that the network: (i) had scale free property indicating that it follows the role that “riches get richer”; and (ii) had the tendency to contain HUB nodes with the maximum load placed for TP53 (tumor protein p53).

Through statistical analysis we identified the following eight HUB nodes: RXRA, VEGFA, NR3C1, PDE4D, MAP3K7, TP53, PHGDH and NDRG1. Moreover, also in the case of the kidney cancer, to define the related HUB – HUB interaction sub-network, we extracted the interactions between HUB nodes and demonstrated that this sub-network is composed of 386 nodes and 460 interactions.

However, we performed also a protein-protein network analysis by STRING tool (https://string-db.org/) to verify that three hundred and thirteen proteins modulated by the arsenicals in KiC are really correlated between them by physical interactions and/or regulatory effects, experimentally proven. This network showed that about 6% (corresponding to twenty-one proteins) and 23% (corresponding to seventy-two proteins) out the three hundred and thirteen proteins had no relation with confidence values of 0.4 and 0.7, respectively. Certainly, these results confirmed that the three hundred and thirteen proteins modulated by the arsenicals in KiC are correlated between them as suggested also by the significant p-values in the functional analysis (Supplementary Fig. 5 and Supplementary Table 5).

The mutational analysis performed by using TCGA_PRAD dataset on the eight HUB nodes showed the presence of mutations in all of them (RXRA, VEGFA, MAP3K7, TP53, PHDGH, NDRG1, NR3C1 and PDE4D) with frequency percentages ranging from 1% to 18% (Table 1). Notably, the most altered nodes are TP53 and MAP3K7, confirming their role in PrC development.

### Identification and analysis of the linking sub-network between Kidney, Prostate and Bladder cancer networks

**Figure 1:**
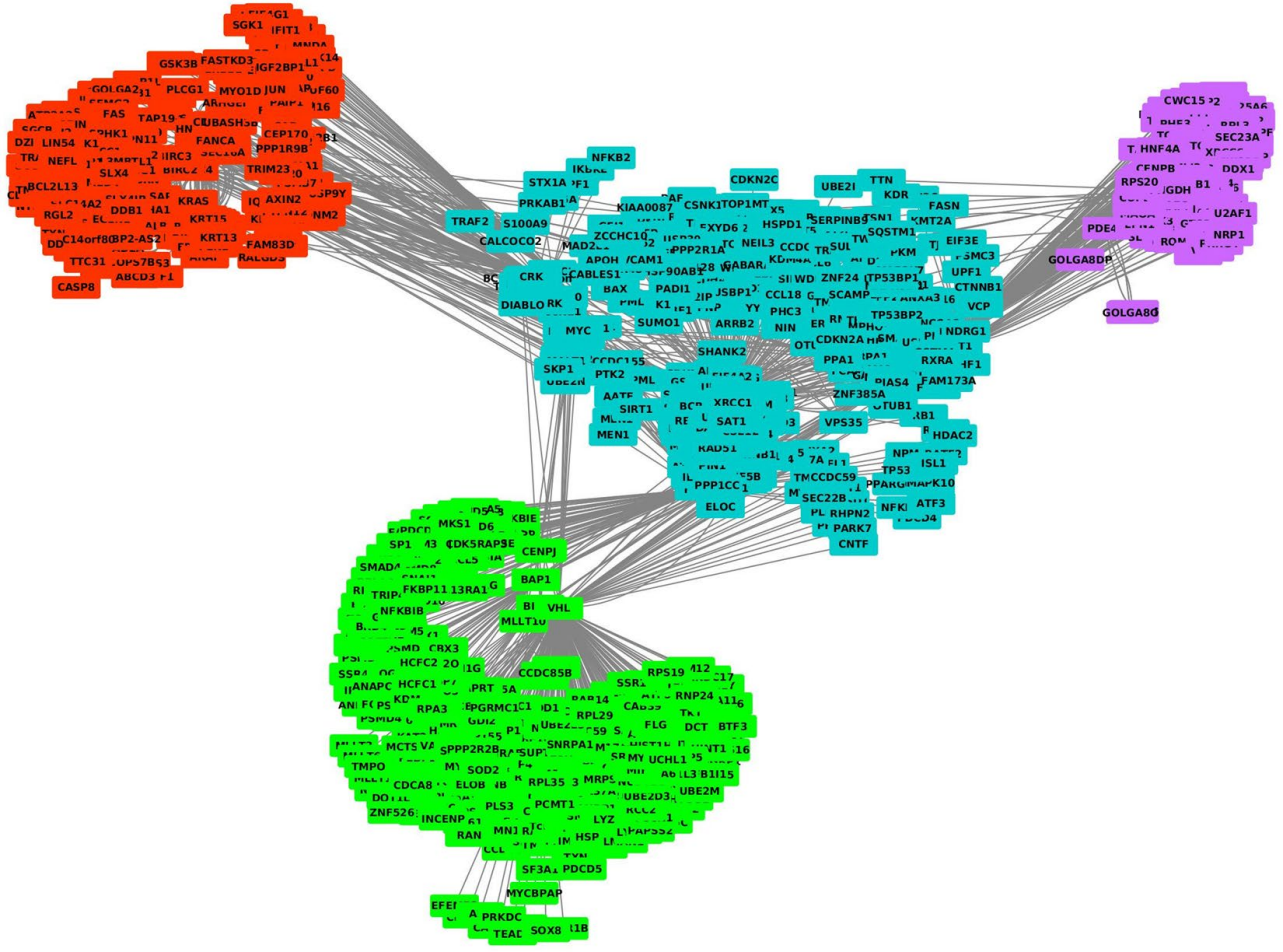
First order interaction network obtained by merging three networks related to proteins modulated by arsenicals in Bladder, Kidney and Prostate cancer. We report the nodes of the Bladder cancer network in red, the nodes of Kidney cancer network in green, the nodes of Prostate cancer network in purple and the nodes linking the three networks in green water.

Since kidney, prostate and bladder are part of the urinary system and all three cancers are subjected to arsenicals exposure, we have merged and analyzed the networks of kidney and prostate with the network reported in our recent work for BlC^20^, in order to find the nodes linking these three cancers. In particular, we have merged the HUB-HUB interaction sub-networks related to the three cancers, and obtained a merged network composed of 942 nodes and 1273 interactions. The detailed analysis of this network evidenced the presence of four sub-networks of which one composed of 199 nodes specific for the involvement of the arsenicals in bladder cancer (reported in red in Fig. 1); one composed of 278 nodes specific for the involvement of the arsenicals in kidney cancer (reported in green in Fig. 1); one composed of 122 nodes specific for the involvement of the arsenicals in prostate cancer (reported in purple in Fig. 1), and one composed of 343 nodes linking the three sub-networks (reported in green water in Fig. 1).

Focusing on these 343 linking nodes we performed a network analysis with the same method described before and accordingly to the previously described parameters. The obtained network has a centralization value of 0.217, a network density of 0.002, a heterogeneity value of 4.638, a characteristic path length of 4.091, a clustering coefficient of 0.022 and an average number of neighbors of 2.291 (Table 2). Moreover, the plots of the node degree distribution, clustering coefficient, stress centrality, closeness centrality and betweenness centrality^23–26^ (Supplementary Fig. 6) confirmed that the network: (i) had scale free property indicating that it follows the role that “riches get richer”; and (ii) had the tendency to contain HUB nodes.

In fact, the statistical analysis based on centrality and topological measures evidenced the presence of seven nodes that can be defined as HUB nodes: RXRA, MAP3K7, NR3C1, PABPC1, NDRG1, RELA and CTNNB1. It is important to underline that RXRA, MAP3K7, NR3C1 and NDRG1were found as HUB nodes in PrC network, RELA in KiC network and PABPC1 in BlC network^20^. On the other hand, CTNNB1 resulted as a new HUB node in this analysis suggesting that it plays an important role in linking the three cancer networks and, hence, could be involved in the arsenicals- induced cancer development and progression (Fig. 1).

**Figure 2:**
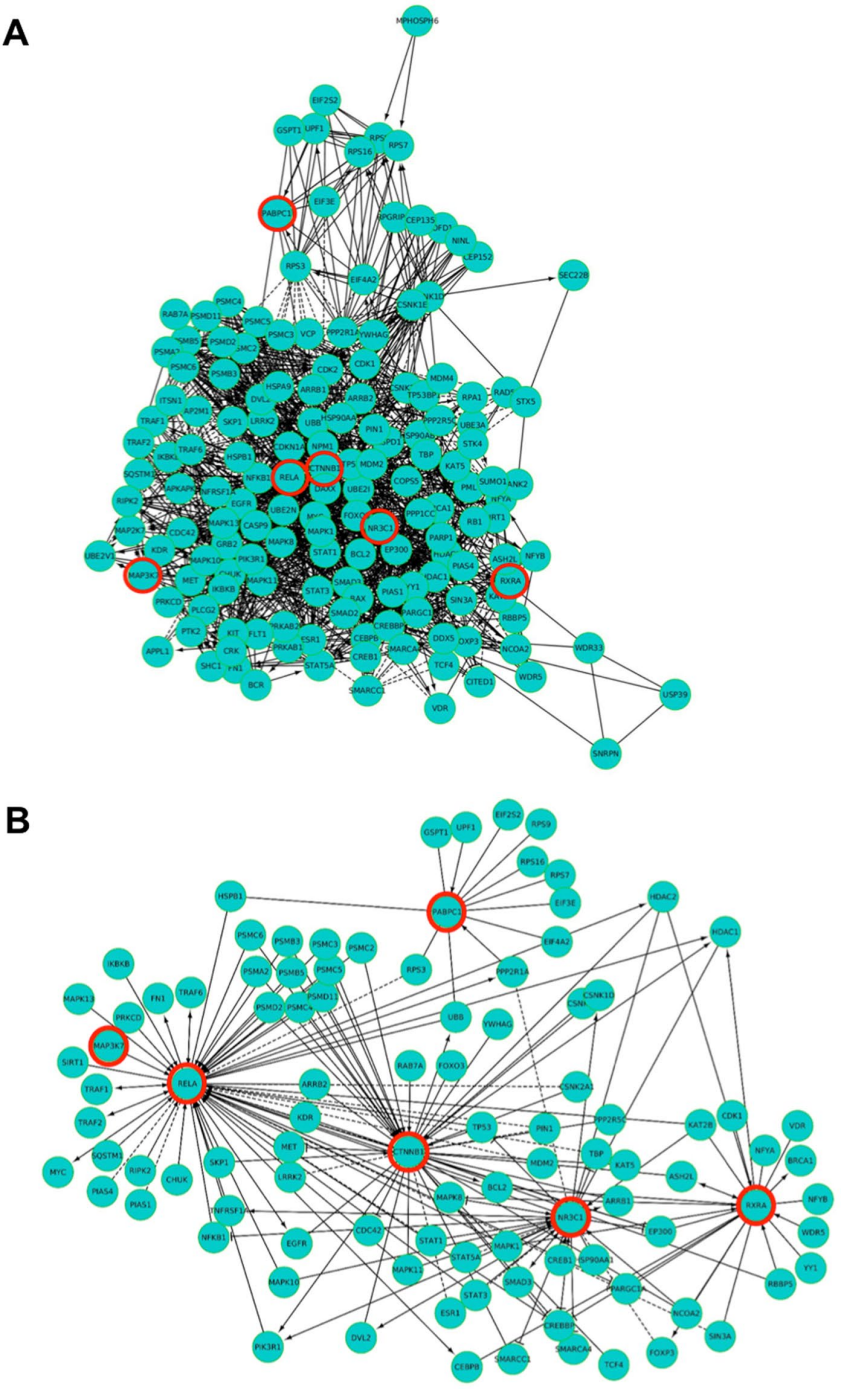
(**A**) Functional interaction network of 156 linking nodes. (**B**) Detailed functional interactions between RELA, RXRA, CTNNB1, NR3C1, MAP3K7 and PABPC1 HUB nodes (evidenced by red circles) of linking zone.

Moreover, a functional analysis was conducted by mapping the 343 linking nodes on the human reactome, to elucidate the molecular functional relationships between them as well as between the seven HUB nodes. From our analysis, 156 out of 343 nodes showed a functional interaction. In detail, a total number of 1570 functional interactions was identified between them (Fig. 2A). Focusing on HUB nodes we can underline that: (i) MAP3K7 activates RELA; (ii) NR3C1 and RELA belong to the same complex; (iii) for the other HUB nodes we observed an indirect relationships between them, highlighting a complex network of reactions with several other molecules, including 18 inhibitions and 77 activations, and 64 complexes (Fig. 2B). These data are useful to understand the type of functional relationships existing between the nodes that link the three cancers and their development upon arsenical exposure.

To verify if the nodes belonging to the central region of the merged network are mutated in the three cancers, we performed a mutational status analysis on the 343 identified nodes. Our analysis evidenced that there are 200 mutated nodes in BlC, 181 in KiC and 189 in PrC (Supplementary Table 6). In detail, 34, 27 and 18 nodes are mutated only in BlC, KiC and PrC, respectively. On the other hand, for 53 nodes there are no data about their mutational status within the three cancers in the corresponding datasets used in our analysis. However, focusing on seven HUB nodes resulted to be important in the central region of the merged network, we evidenced that all of them are reported as mutated: (i) in BlC with frequency percentages ranging from 1.2% to 17%; (ii) in KiC with frequency percentages ranging from 0.4% to 14%; (iii) in PrC with frequency percentages ranging from 1.2% to 15% (Table 3). The most altered nodes among them are MAP3K7, NR3C1, PABPC1 and CTNNB1 with mutation frequencies equal to 15% in PrC, 14% in KiC, 17% in BlC and 11% in KiC, respectively.

**Table 3:** Mutational status of HUB nodes in the networks obtained for proteins modulated by arsenicals in the linking sub-network.

HUB	Linking sub-network		
	BlC	KiC	PrC
RXRA	5%	0.4%	1.6%
MAP3K7	2.7%	0.4%	15%
NR3C1	1.2%	14%	1.2%
PABPC1	17%	4%	7%
NDRG1	7%	1.1%	7%
RELA	3%	0.9%	2.2%
CTNNB1	4%	11%	3%

**Figure 3:**
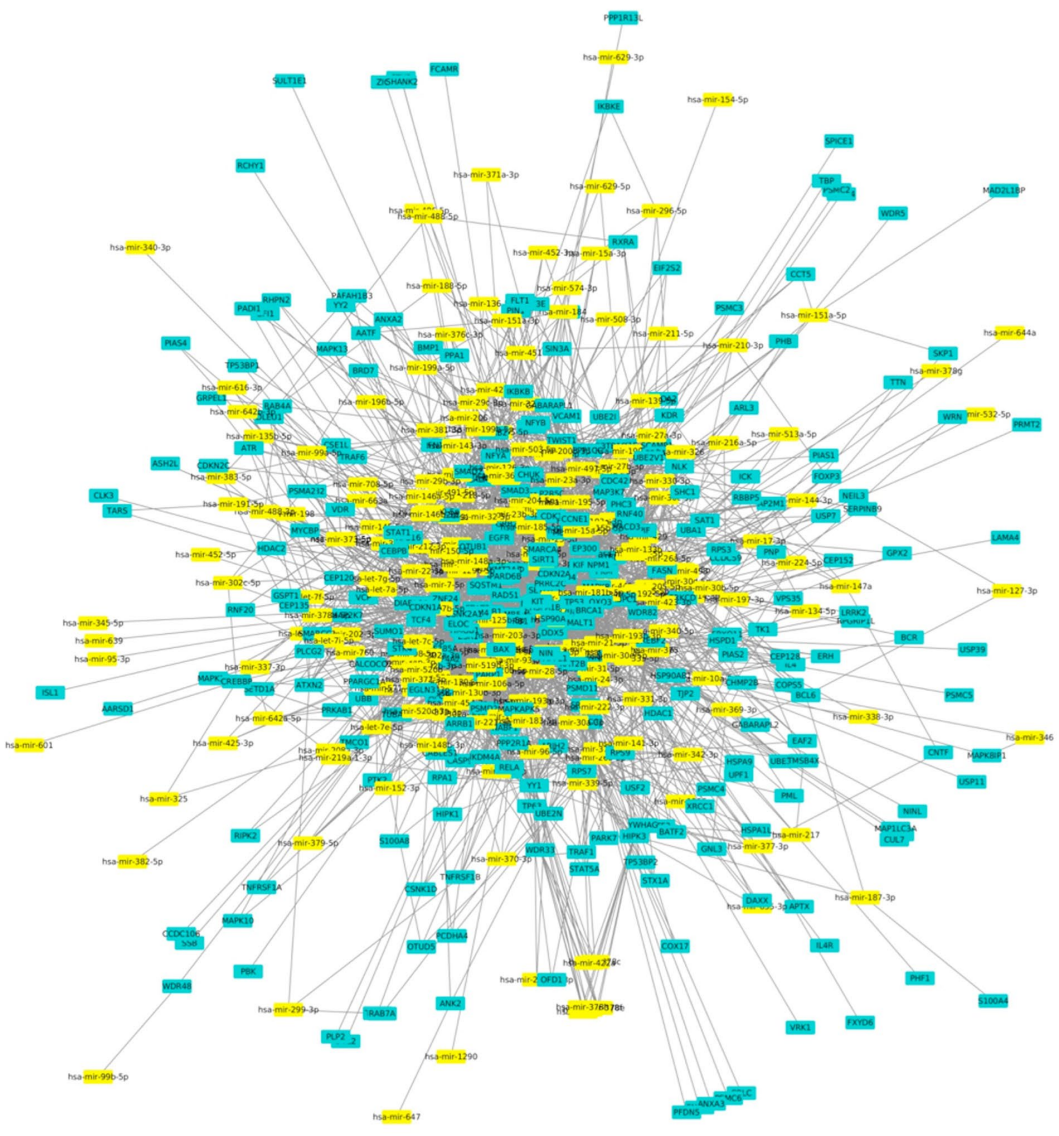
miRNAs targeting nodes in linking region of merged network. In detail, nodes are represented in green water while miRNAs are displayed in yellow.

Furthermore, to understand if the 343 nodes that link PrC, KiC and BlC networks can be targeted by miRNAs, we performed an integrated analysis as described in the Methods section. In detail, we found that 233 miRNAs are able to target these 343 nodes. Thus, we obtained an interaction network between miRNAs and the 343 nodes characterized from 2755 interactions (Fig. 3).

Regarding the seven HUB nodes, they are targeted by a total of 73 miRNAs. In detail, 10 miRNAs targeted two or three HUB nodes whereas 3, 10, 20, 10, 8, 5 and 7 miRNAs resulted able to target only one HUB node such as RXRA, MAP3K7, NR3C1, PABPC1, NDRG1, RELA and CTNNB1, respectively (Table 4).

**Table 4:** miRNAs able to target the seven HUB nodes in the linking sub-network.

miRNA	HUB
hsa-mir-34a-5p	CTNNB1, MAP3K7, NDRG1
hsa-mir-423-3p	PABPC1, RXRA
hsa-mir-10a-5p	MAP3K7, PABPC1
hsa-mir-34b-5p	CTNNB1, MAP3K7
hsa-mir-182-5p, hsa-mir-19b-3p	NDRG1, NR3C1
hsa-mir-155-5p	CTNNB1, NR3C1
hsa-let-7b-5p	NDRG1, PABPC1
hsa-mir-30a-5p	CTNNB1, PABPC1
hsa-mir-320a	CTNNB1, RELA
hsa-mir-574-3p, hsa-mir-488-5p, hsa-mir-27a-3p	RXRA
hsa-mir-497-5p, hsa-mir-16-5p, hsa-mir-143-3p, hsa-mir-15a-5p, hsa-mir-646, hsa-mir-107, hsa-mir-195-5p, hsa-mir-503-5p, hsa-mir-424-5p, hsa-mir-15b-5p	MAP3K7
hsa-mir-22-3p, hsa-mir-130b-3p, hsa-mir-17-5p, hsa-mir-106b-5p, hsa-mir-126-5p, hsa-mir-32-5p, hsa-mir-18a-5p, hsa-mir-137, hsa-mir-519d-3p, hsa-mir-106a-5p, hsa-mir-144-3p, hsa-mir-204-5p, hsa-mir-377-3p, hsa-mir-211-5p, hsa-mir-93-5p, hsa-mir-20a-5p, hsa-mir-20b-5p, hsa-mir-183-5p, hsa-mir-374a-5p, hsa-mir-369-3p,	NR3C1
hsa-mir-200b-3p, hsa-mir-193b-3p, hsa-mir-429, hsa-mir-200c-3p, hsa-mir-125b-5p, hsa-mir-149-5p, hsa-mir-17-3p, hsa-mir-10b-5p, hsa-mir-1256, hsa-mir-34c-5p	PABPC1
hsa-mir-148a-3p, hsa-mir-24-3p, hsa-mir-19a-3p, hsa-mir-1290, hsa-mir-335-5p, hsa-mir-148b-3p, hsa-mir-152-3p, hsa-mir-342-3p	NDRG1
hsa-mir-7-5p, hsa-mir-30e-5p, hsa-mir-373-3p, hsa-mir-96-5p, hsa-mir-186-5p	RELA
hsa-mir-375, hsa-mir-101-3p, hsa-mir-221-3p, hsa-mir-214-3p, hsa-mir-331-3p, hsa-mir-370-3p, hsa-mir-200a-3p	CTNNB1

All these data suggest that miRNAs can have an important role to influence the modulation of the HUB nodes and that further studies can be useful to explain their possible role in the arsenical involvement in PrC, KiC and BlC development.

## Discussion

Considering that cancers do not result from a single mutation or gene/protein but a combination of perturbed genes/proteins acting in molecular networks that correspond to hallmark processes such as apoptosis and cell proliferation, the network analysis represents a simple and efficient method to model biological systems. In a network, the nodes or vertices represent genes or proteins or miRNAs while the edges show their functional or physical interactions. The systems biology of the cellular network is very similar to a roadmap where a driver finds alternate routes to arrive at a specific destination if there is car accident that block a road. In the same way, in a cell if a protein is compromised in a signaling network, the entire function of the cell is modified resulting in a disease phenotype including cancer^27–30^. For this reason, the network analysis has been recently used to explain different mechanisms correlated to cancer. For example, the use of network approach has been suggested as useful: (i) for cancer intervention through the targeting of key phosphotyrosine sites and their associated signaling hubs in the network^27^; (ii) to predict breast cancer subtype-specific drug targets^28^; (iii) to suggest tumor clinical phenotypes using genome sequencing data^29^; and (iv) to identify that the feed-forward loop - FFL (PDGF/FLT1/SHC1) is significantly enriched in the PIK3CA-mutated luminal-A tumor patients and predicts survival outcome^30^.

In this our study we used a network analysis approach to highlight the correlation between genes and proteins that are modulated by the arsenicals in KiC and PrC and to integrate the identified networks with that obtained in our recent work on the correlation between arsenicals and BlC^20^.

**Figure 4:**
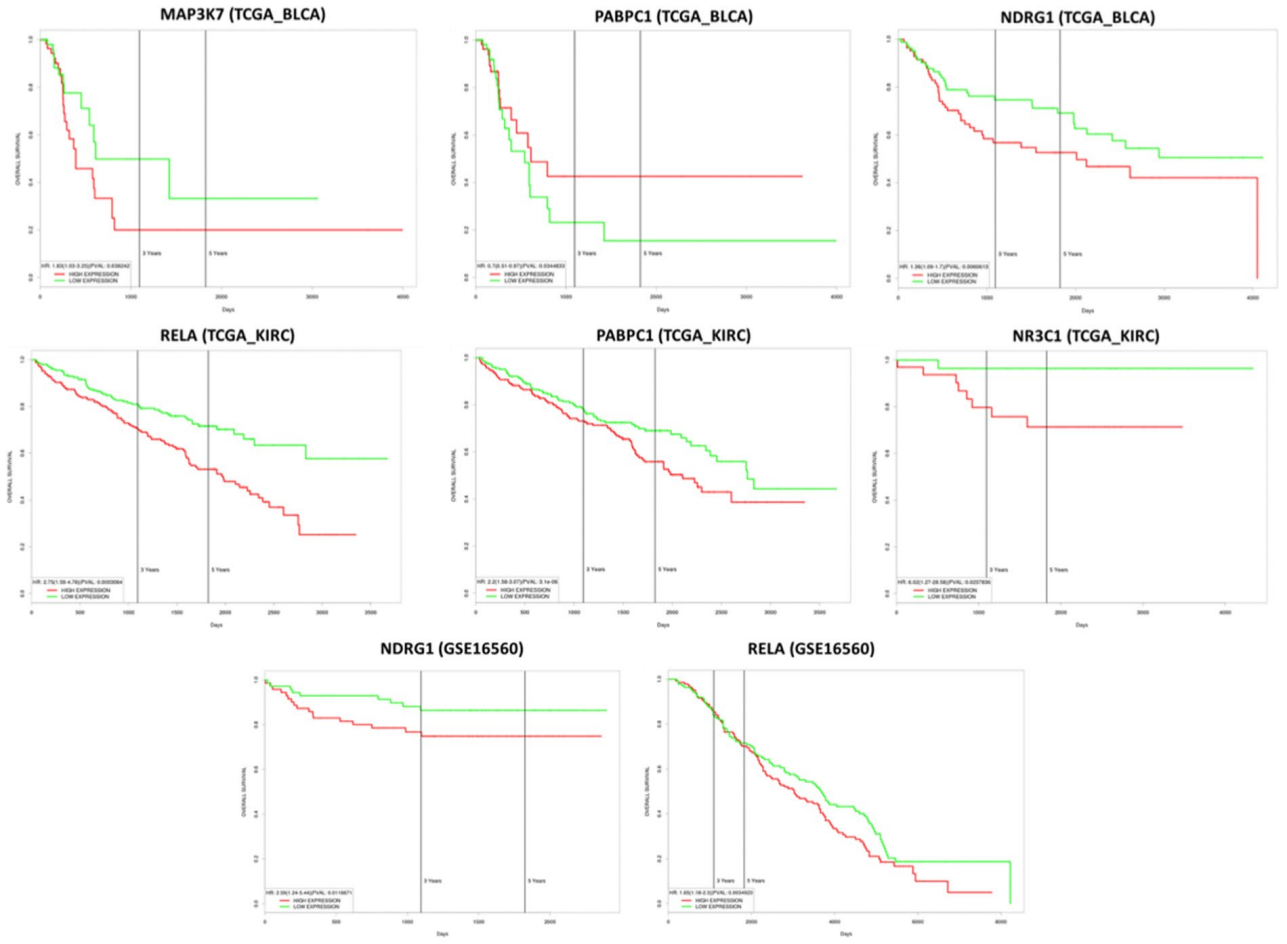
Evaluation of the Kaplan-Meyer survival curves of HUBs, that link PrC, BlC and KiC networks, associated to poor survival of patients with three cancers.

The analysis of our networks has evidenced: (i) nine HUBs (BAP1, RELA, TP53, BIRC5, VHL, IL4R, MLLT10 and PIDD1) specific for “arsenicals and KiC” network; (ii) nine HUBs (RXRA, VEGFA, NR3C1, PDE4D, MAP3K7, TP53 and NDRG1) specific for “arsenicals and PrC” network; (iii) TP53 in common between two networks. These two networks were compared with BlC network including seven HUBs (BIRC3, CCNE1, ERCC4, FANCA, PABPC1, PRSS3, PSMB2) nodes^20^. To find a possible link between these three cancers subjected to arsenicals exposure, we merged the three networks and identified seven HUB nodes (RXRA, MAP3K7, NR3C1, PABPC1, NDRG1, RELA and CTNNB1) of the linking region between three cancer networks. To examine if these HUBs were associated to poor survival of patients with BlC, KiC and PrC we evaluated the Kaplan-Meyer survival curves (Supplementary Table 7 and Fig. 4). This analysis evidenced that: (i) down-expression of PABPC1 is associated with poor survival of BlC patients; (ii) high expression of MAP3K7 and NDRG1 is associated with poor survival of BlC patients; (iii) high expression of NR3C1, RELA and PABPC1 is associated with poor survival of KiC patients; (iv) high expression of NDRG1 and RELA is associated with poor survival of PrC patients.

**Figure 5:**
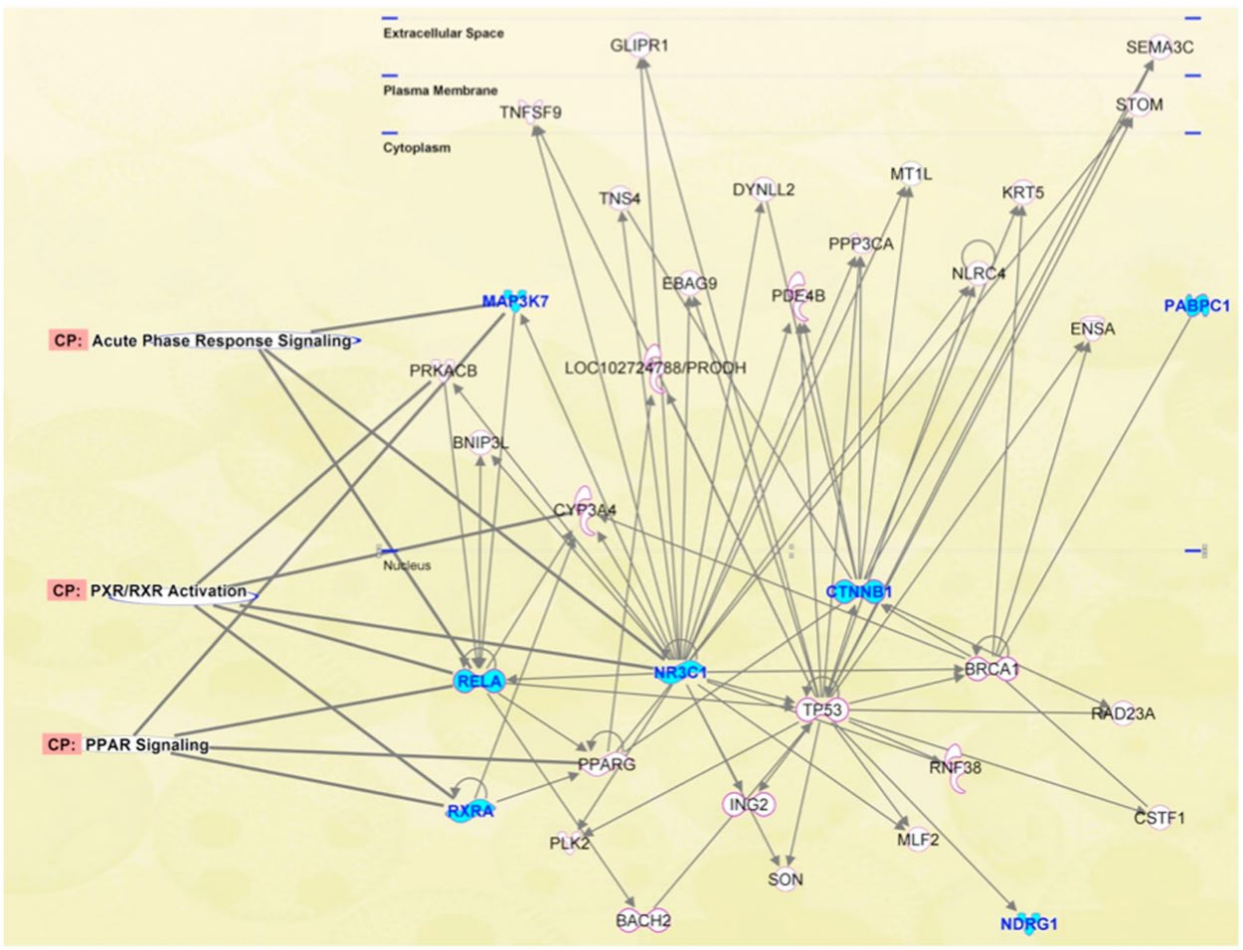
Ingenuity Pathway Analysis (IPA) conducted on the seven HUB nodes (RXRA, MAP3K7, NR3C1, PABPC1, NDRG1, RELA and CTNNB1) shown by cyan symbols. Other nodes are reported by white symbols. The molecular pathways are indicated on the left of the network.

Ingenuity Pathway Analysis (IPA) was conducted on these seven HUB nodes to verify if they are correlated between them and are involved in common molecular pathways. This analysis evidenced that RXRA, MAP3K7, NR3C1, PABPC1, NDRG1, RELA and CTNNB1 are present in the same network that shows also the presence of other important nodes such as TP53 and PPARG (Fig. 5). In details, TP53 is correlated with CTNNB1, NR3C1, RELA and NDRG1, and PPARG with RXRA, RELA and NR3C1. Moreover, this analysis has also demonstrated that MAP3K7, RELA and NR3C1 are involved in acute phase response signaling, RELA and NR3C1 in RXR activation, and RXRA and RELA in PPAR signaling.

Several of the identified nodes in the single cancer were reported in the TCGA datasets as mutated, corroborating the validity of our approach and confirming their potential central role in cancer development. Moreover, among those nodes identified in KiC, it has been already reported that: (i) VHL and BAP1 are essentials for kidney functions and their somatic mutations are essential in kidney tumorogenesis^31^; (ii) mutations of IL4R are associated with development of KiC^32^; (iii) the increased p53 expression in KiC occurs even if it is not mutated^33^. Similarly for those identified in PrC, it has been already reported that mutations in TP53 and PDE4D correlated with prostate cancer metastatic progression^34^.

Notably, for all the seven HUB nodes of the linking region between three cancer networks, mutations were found in all three cancers (BlC, KiC, PrC) with the higher frequency percentages identified for four nodes: MAP3K7, NR3C1, PABPC1 and CTNNB1. Mutations of MAP3K7 are known to promote aggressive PrC^35^ while mutations of RXRA are associated with the activation of peroxisome proliferator-activated receptors (PPAR) signaling pathway, leading to the urothelial proliferation in PrC and BlC^36,37^. Hence, our data confirm that all seven nodes of the linking region between three cancer networks (RXRA, MAP3K7, NR3C1, PABPC1, NDRG1, RELA and CTNNB1) have a role in the cancerogenesis of BlC, KiC and PrC and suggest that they can be used as biomarker to improve the diagnosis and prognosis of these three cancers.

Indeed, several data in the literature reported the altered expression and the involvement of these seven molecules in cancers and, particularly, in BlC, PrC and KiC. In detail, NDRG1 mRNA levels resulted higher in BlC, PrC and KiC and associated with invasiveness, metastasis and recurrence of these cancers^38–40^ and down-regulated in the presence of arsenic trioxide^41^. RELA is constitutively activated in PrC cells^42^, has an increased activation in KiC^43^, representing a possible therapeutic target in this cancer^44^, and is increased in the presence of arsenicals^45^. RXRA in KiC has been suggested as an independent predictor of poor survival^46^ and its inactivation in the prostate epithelium resulted in the development of preneoplastic lesions^47^. However, it is important to underline that arsenite trioxide has been reported to induce the phosphorylation of RXRA, subsequent to oxidative damages and the activation of the stress-activated protein kinases cascade^48^. MAP3K7 was reported to be down-regulated in BlC and PrC^49^. NR3C1 resulted to be down-regulated in KiC as well as in BlC^50,51^ and its NR3C1 polymorphism was associated to PrC risk^52^. PABPC1 is involved in cytoplasmic regulatory processes of mRNA metabolism such as pre-mRNA. PABPC1 levels were significantly increased in BlC^53^ and its up-regulation in PrC tissue has been reported to be correlates to increased recurrence^54^. CTNNB1 expression was found to be up-regulated in KiC and its knockdown inhibited cell proliferation, migration, and invasion and induced apoptosis of KiC cells, suggesting that can be considered a potential therapeutic target in this disease^55^. Moreover CTNNB1 up-regulation and polymorphism have been associated to BlC and PrC risk^56,57^.

Moreover, considering that these seven HUB nodes are present in robust combinatory cancer hallmark-based gene signature sets that more accurately predict prognosis^58^, we decided also to search the cancer related miRNAs able to target these nodes. Our analysis has also evidenced that the seven HUBs linking three PrC, BlC and KiC networks are targeted by 10 miRNAs (hsa-mir-34a-5p, hsa-mir-423-3p, hsa-mir-10a-5p, hsa-mir-34b-5p, hsa-mir-182-5p, hsa-mir-19b-3p, hsa-mir-155-5p, hsa-let-7b-5p, hsa-mir-30a-5p, hsa-mir-320a). Interestingly, several evidences correlated these miRNAs with PrC, BlC and KiC. In details, hsa-mir-34a-5p by regulating stathmin-1 oncoprotein was reported to inhibit proliferation and progression in PrC^59,60^ as well as the cellular invasion in BlC^61^. Low expression rate of hsa-mir-34a-5p was reported to correlate with the malignancy and tumor size of BlC^62^. Li *et al*.^63^ showed that hsa-mir-34a-5p suppressed cellular growth in KiC and the metastasis formation. Low levels of hsa-mir-34b-5p represent a robust biomarker for PrC progression^64^ and its polymorphism increases the risk of PrC^65^. This miRNA suppresses the cellular proliferation in BlC and is downregulated in KiC cells^66^ and in BlC^67^. Hsa-mir-423-3p polymorphism is associated with increased BlC risk^68^. Hsa-mir-10a-5p is indicated as a candidate BlC biomarkers^69^, is up-regulated in PrC tissues compared to their own normal tissues and down-regulated in KiC^70^. Hsa-mir-30a-5p is down-regulated in BlC and PrC^71,72^. Moreover, the expression levels of miR-30a-5p in KiC cells were demonstrated to be significantly downregulated and suggested to have a tumor-suppressive role in the tumorigenesis of kidney cancer^73^. Hsa-mir-182-5p is associated with growth, migration and invasion in PrC via suppression of FOXO1^73^ and is resulted to be upregulated also in BlC^74^. On the other hand, this miRNA is frequently down-regulated in KiC tissues and its restoration was considered as a potential therapeutic strategy for KiC therapy^75^. The up-regulation of hsa-mir-19b-3p demonstrated highest diagnostic sensitivity and specificity in PrC^76^. It has been found that hsa-mir-19b-3p is highly expressed in BlC^77^ and its enrichment has been described as being directly associated with KiC^78^. About hsa-mir-155-5p it is necessary to underline that its expression was upregulated in PrC tissues and cell lines^79^, in KiC^80^ and in BlC tissues by reducing the expression of the tumor suppressor DMTF1^81^. It has been reported that hsa-let-7b-5p is up-regulated in BlC^82^ whereas its expression was significantly decreased in KiC tissues and its dysregulation has been associated with pathological grade^83^. This miRNA enhances tumor-associated macrophages to promote angiogenesis and mobility in PrC^84^. Finally, aberrantly expressed hsa-mir-320a contributes to BlC cells invasion through directly down-regulating ITGB3 protein expression in BlC^85^. On the other hand, this miRNA resulted to be significantly reduced in PrC tissues, suggesting that it may be a promising anticancer miRNA^86^. Moreover, hsa-mir-320a is a direct regulator of Aquoporin 1 and 4, which are integral membrane transporters involved in water homeostasis and is critical regulator of kidney function and homeostasis maintenance^87^.

Only few data are available about the expression changes of these ten miRNAs upon arsenical exposure and the molecular mechanisms of arsenical-induced toxicity. For example, the expression of hsa-mir-10a-5p, hsa-mir-182-5p and hsa-mir-19b-3p resulted to be up-regulated in HUVEC cells treated with 20 μM of arsenite^88^. On the other hand, arsenical exposure was able to induce the reduction of hsa-mir-34a-5p and let-7b and caused cumulative disruption to epigenetic regulation of hsa-mir-34a-5p expression in keratinocytes^89^. Moreover, hsa-mir-30a-5p is resulted to be up-regulated after arsenic trioxide treatment in liver cancer cells^88^ whereas arsenical exposure significantly down-regulated the expression of hsa-mir-423-3p in rat liver tissues^90^.

In conclusion, our interaction network analysis has showed what are the genes/proteins/miRNAs that link three BlC, KiC and PrC cancers to the exposure to arsenicals and higlight the necessity to study in more detail the molecular mechanisms of arsenical-induced toxicity.

## Methods

### Network analysis

The list of molecules belonging to arsenical family with the related proteins known to be modulated from them in kidney and prostate cancers was extracted by CTD^91^. Cytoscape platform (http://www.cytoscape.org/) was used to create the interaction networks between the selected proteins using the human molecular interactome (INTACT) as ref.^21^. Some statistical analyses based on measures of centrality and topology were used to identify the nodes with a large degree and have connections with many other nodes in the networks defined as HUB nodes^20,92–95^.

Functional and Pathway Analyses were performed by DAVID program^96^.

We also interrogated the reactome database (www.reactome.org) that links the proteins to their molecular functions and provides information on the related biological reactions at molecular level and groups the reactions that belong to a higher order process. The reaction term refers to the description of any event in biological processes such as biochemical reactions, transport reactions, post- translational modifications, macromolecules assembly and so on^97^. Hence, this database is useful to store the information regarding the biological processes and to discover new insight in unexpected protein functional relationships^98^. It is important to underline that the term functional interaction refers to an interaction in which two proteins are involved in the same biological reaction such as catalysis, activation, inhibition or as members of the same protein complex. We have used the ReactomeFIViz app in Cytoscape program to construct network by extracting interactions from reactions and complexes of human annotated pathways and to find the network functional interaction patterns in the merged network of prostate, kidney and bladder cancers. In detail, we have built and functionally annotated the reactome network, and, then, extracted the network modules of highly-interacting groups of proteins.

### miRNA evaluations

The list of miRNAs known to be dysregulated in PrC and KiC were selected by MirNet tool^99^ using the same protocol applied to BlC in our recent paper^20^. Starting from them we extracted all their targets and selected the miRNAs able to target our HUB nodes. Finally, we constructed the interaction networks between miRNAs and HUB nodes for kidney and prostate cancers, and compared these two networks with that of BlC^20^ in order to identify common interacting miRNAs/HUB nodes between the three networks.

### Mutational status evaluations

In order to test the presence of mutations on the identified genes involved in the three cancers we analyzed three TCGA datasets (TCGA_BLCA, TCGA_KIRC and TCGA_PRAD) using CbioPortal tool (http://cbioportal.org)^100,101^.

### Kaplan-Meyer survival analysis

Kaplan-Meyer survival analysis has been applied to examine the association of genes/pathways with poor patient survival by PROGgeneV2 tool (http://watson.compbio.iupui.edu/chirayu/proggene/database/index.php)^102,103^. This is a web application that can be used for studying prognostic implications of mRNA biomarkers in 18 cancer types by using public repositories such as GEO, EBI Array Express and The Cancer Genome Atlas. For all other cancers this tool has one or more of overall, metastasis free and recurrence free survival variables. Survival calculations were done using R library ‘Survival’ which is also coded in the backend. In each dataset, for the selected gene, survival information in terms of survival status (overall or metastasis free or recurrence free survival), and survival time (time to death or time to metastasis or time to recurrence) are retrieved along with gene expression as continuous variable. Using median gene expression value as bifurcating point, samples are divided into High and Low gene expression groups. Using survival data and continuous expression variable, survival analysis is done by fitting cox proportional hazards model using function “coxph” of library survival. Hazard ratio (HR) as ‘exp (coef)’ and log rank p value are retrieved from the fitted model. To create prognostic plot, High and Low expression categorical variable is used along with survival data. Plots are created using function ‘survfit’ of the same R library. Final plots, which show survival in High and Low expression arms of samples, annotated for HR, HR confidence intervals and p value are exported as images.

## Electronic supplementary material

Supplementary Tables 1-7 and Figures 1-6
