# Supplementary material for: Identifying a panel of genes/proteins/miRNAs modulated by arsenicals in bladder, prostate, kidney cancers
Source: Sci Rep. 2018 Jul 10;8:10395. doi: 10.1038/s41598-018-28739-6 (PMC6039466; doi:10.1038/s41598-018-28739-6)
Supplement: Supplementary file 1 — Supplementary Tables 1-7 and Figures 1-6 [file 41598_2018_28739_MOESM1_ESM.docx]

**Identifying a panel of genes/proteins/miRNAs modulated by arsenicals in bladder, prostate, kidney cancers**

Andrea Polo^1^, Silvia Marchese^1^, Giuseppina De Petro^2^, Maurizio Montella^3^, Gennaro Ciliberto^4^, Alfredo Budillon^1*^, Susan Costantini^1*^

*^1^ Experimental Pharmacology Unit, IRCCS Istituto Nazionale Tumori “Fondazione Giovanni Pascale”, Napoli, Italia*

*^2^ Dipartimento di Medicina Molecolare e Traslazionale, Università di Brescia, Brescia, Italia*

*^3^ Unità di Epidemiologia*, *IRCCS Istituto Nazionale Tumori “Fondazione G. Pascale”, Napoli, Italia*

*^4^Scientific Directorate, IRCCS Istituto Nazionale Tumori “Regina Elena”, Roma, Italia*

*** Corresponding Authors:**

Dr. Alfredo Budillon

Istituto Nazionale Tumori “Fondazione G. Pascale”

Via Mariano Semmola

80131 Napoli

Tel.: +39 081-5903202

E-Mail: a.budillon@istitutotumori.na.it

Dr. Susan Costantini

Istituto Nazionale Tumori “Fondazione G. Pascale”

Via Ammiraglio Bianco

83013 Mercogliano (Av)

E-Mail: s.costantini@istitutotumori.na.it

Tel.: +39 0825-1911729; Fax: +39-0825-1911705.

**SUPPLEMENTARY MATERIAL**

**Supplementary Table 1**. **Arsenicals implicated in bladder, kidney and prostate cancer.** We reported the list of the arsenicals, the compound type and the number of modulated proteins for each compound in BlC, KiC and PrC.

|  | | **Modulated Proteins** | | |
| --- | --- | --- | --- | --- |
| **CHEMICALS** | **COMPOUND TYPE** | **BlC** | **KiC** | **PrC** |
| monomethylarsonous acid | INORGANIC | 27 | 22 | 74 |
| arsenite | INORGANIC | 38 | 34 | 121 |
| arsenic | METAL | 46 | 57 | 168 |
| sodium arsenite | INORGANIC | 40 | 66 | 223 |
| arsenic acid | INORGANIC | 17 | 15 | 50 |
| cacodylic acid | ORGANIC | 6 | 5 | 11 |
| 4-aminophenylarsenoxide | INORGANIC | 5 | 6 | 10 |
| galliumarsenide | INORGANIC | 1 | 4 | 7 |

**Supplementary Table 2**. Total list of proteins modulated by arsenicals in kidney cancer

| GSTT1 | ACHE | HARS |
| --- | --- | --- |
| APAF1 | ALK | PRCC |
| IL6 | ANXA4 | VHL |
| BAP1 | CAPG | CRABP1 |
| CCND1 | CASP2 | TNFSF10 |
| CDKN1B | CSMD3 | TSC2 |
| CYP1A1 | CTSD | GJB1 |
| EIF4EBP1 | DIRC2 | BIRC7 |
| IL2 | ELOC |  |
| MAPK1 | FMN2 |  |
| MAPK3 | IL4R |  |
| PTGS2 | IL6R |  |
| SOD2 | KCNMA1 |  |
| CARD11 | KDM5C |  |
| CRYAB | MLLT10 |  |
| ERBB2 | MTOR |  |
| HSPA9 | NLRP12 |  |
| HSPB1 | PGK1 |  |
| IL13 | PIDD1 |  |
| KEAP1 | SCARB1 |  |
| KRT7 | TET2 |  |
| NAV3 | YIPF3 |  |
| RELA | NDRG1 |  |
| TP53 | APRT |  |
| AURKA | CRADD |  |
| TNFRSF9 | CTSB |  |
| AHNAK | DNHD1 |  |
| ALAD | EEF2 |  |
| ALDH1A1 | EPAS1 |  |
| BIRC5 | FAM111B |  |
| DAPK1 | FLT1 |  |
| GSTM1 | HNF1A |  |
| GSTP1 | KRT32 |  |
| HNF1B | L1CAM |  |
| HSPD1 | LDHB |  |
| KRT8 | MET |  |
| OGG1 | PDHB |  |
| PTEN | PEBP1 |  |
| SFRP2 | PIK3CA |  |
| SLC2A1 | TSC1 |  |
| ABCB1 | ZNF804A |  |
| BRAF | GSTT1 |  |
| IL32 | MAPK1 |  |
| RAF1 | PTGS2 |  |

**Supplementary Table 3**. Molecular pathways in which ninety-six proteins are involved in kidney cancer with the relative p-value scores (p values < 0.05 are considered as significant).

| **PATHWAYS** | **PROTEINS** | **p-value** |
| --- | --- | --- |
| HIF-1 signaling pathway | RELA, CDKN1B, ERBB2, EIF4EBP1, FLT1, IL6R, IL6, MTOR, MAPK1, MAPK3, PIK3CA, PDHB, SLC2A1, VHL | 4.6e-12 |
| Pathways in cancer | BRAF, MET, RELA, RAF1, BIRC5, BIRC7, CCND1, CDKN1B, DAPK1, EPAS1, ERBB2, IL6, MTOR, MAPK1, MAPK3, PTEN, PIK3CA, PTGS2, SLC2A1, TP53, VHL | 2.8e-10 |
| Central carbon metabolism in cancer | MET, RAF1, ERBB2, MTOR, MAPK1, MAPK3, PTEN, PIK3CA, PDHB, SLC2A1, TP53 | 3.2e-10 |
| PI3K-Akt signaling pathway | MET, RELA, RAF1, CCND1, CDKN1B, EIF4EBP1, FLT1, IL2, IL4R, IL6R, MTOR, MAPK1, MAPK3, PTEN, PIK3CA, TSC1, TSC2, TP53 | 1.8e-9 |
| mTOR signaling pathway | BRAF, EIF4EBP1, MTOR, MAPK1, MAPK3, PTEN, PIK3CA, TSC1, TSC2 | 5.9e-8 |
| Hepatitis B | RELA, RAF1, APAF1, BIRC5, CCND1, CDKN1B, IL6, MPAK1, MAPK3, PTEN, PIK3CA, TP53 | 9.5e-8 |
| FoxO signaling pathway | BRAF, RAF1, CCND1, CDKN1B, IL6, MAPK1, MAPK3, PTEN, PIK3CA, SOD2, TNFSF10 | 4.5e-7 |
| ErbB signaling pathway | BRAF, RAF1, CDKN1B, ERBB2, EIF4EBP1, MTOR, MAPK1, MAPK3, PIK3CA | 1.4e-6 |
| Thyroid hormone signaling pathway | RAF1, CCND1, MTOR, MAPK1, MAPK3, PIK3CA, SLC2A1, TSC2, TP53 | 1.1e-5 |
| Choline metabolism in cancer | RAF1, EIF3EBP1, MTOR, MAPK1, MAPK3, PIK3CA, TSC1, TSC2 | 4.4e-5 |
| Insulin signaling pathway | BRAF, RAF1, EIF4EBP1, MTOR, MAPK1, MAPK3, PIK3CA, TSC1, TSC2 | 4.5e-5 |
| Proteoglycans in cancer | BRAF, MET, RAF1, CCND1, ERBB2, MTOR, MAPK1, MAPK3, PIK3CA, TP53 | 1.1e-4 |
| Focal adhesion | BRAF, MET, RAF1,CCND1, ERBB2, FLT1, MAPK1, MAPK3, PTEN, PIK3CA | 1.3e-4 |
| Sphingolipid signaling pathway | RELA, RAF1, CTSD, MAPK1, MAPK3, PTEN, PIK3CA, TP53 | 1.3e-4 |
| VEGF signaling pathway | RAF1, HSPB1, MAPK1, MAPK3, PIK3CA, PTGS2 | 2.7e-4 |
| p53 signaling pathway | APAF1, CCND1, PIDD1, PTEN, TSC2, TP53 | 4.2e-4 |
| T cell receptor signaling pathway | RELA, RAF1, CARD11, IL2, MAPK1, MAPK3, PIK3CA | 4.2e-4 |
| B cell receptor signaling pathway | RELA, RAF1, CARD11, MAPK1, MAPK3, PIK3CA | 4.8e-4 |
| Prolactin signaling pathway | RELA, RAF1, CCND1, MAPK1, MAPK3, PIK3CA | 5.5e-4 |
| Neurotrophin signaling pathway | BRAF1, RELA, RAF1, MAPK1, MAPK3, PIK3CA, TP53 | 9.4e-4 |
| AMPK signaling pathway | CCND1, EEF2, EIF4EBP1, MTOR, PIK3CA, TSC1, TSC2 | 1.0e-4 |
| MicroRNAs in cancer | ABCB1, MET, RAF1, CCND1, CDKN1B, ERBB2, MTOR, PTEN, PTGS2, TP53 | 1.4e-3 |
| Cytokine-cytokine receptor interaction | MET, TNFRSF9, FLT1, IL13, IL2, IL4R, IL6R, IL6, TNFSF10 | 1.4e-3 |
| Jak-STAT signaling pathway | CCND1, IL13, IL2, IL4R, IL6R, IL6, PIK3CA | 2.5e-3 |
| Apoptosis | RELA, APAF1, PIK3CA, TNFSF10, TP53 | 2.8e-3 |
| TNF signaling pathway | RELA, IL6, MAPK1, MAPK3, PIK3CA, TPGS2 | 3.3e-3 |
| Insulin resistance | RELA, IL6, MTOR, PTEN, PIK3CA, SLC2A1 | 3.6e-3 |
| Oxytocin signaling pathway | RAF1, CCND1, EEF2, MAPK1, MAPK3, PIK3CA, PTGS2 | 3.8e-3 |
| Fc epsilon RI signaling pathway | RAF1, IL13, MAPK1, MAPK3, PIK3CA | 3.9e-3 |
| Transcriptional misregulation in cancer | MET, RELA, CDKN1B, FLT1, IL6, PRCC, TP53 | 5.1e-3 |
| Natural killer cell mediated cytotoxicity | BRAF1, RAF1, MAPK1, MAPK3, PIK3CA, TNFSF10 | 6.0e-3 |
| Chemical carcinogenesis | CYP1A1, GSTM1, GSTP1, GSTT1, PTGS2 | 7.0e-3 |
| Progesterone-mediated oocyte maturation | BRAF, RAF1, MAPK1, MAPK3, PIK3CA | 9.3e-3 |
| Viral carcinogenesis | RELA, CCND1, CDKN1B, MAPK1, MAPK3, PIK3CA, TP53 | 1.3e-2 |
| Rap1 signaling pathway | BRAF, MET, RAF1, FLT1, MAPK1, MAPK3, PIK3CA | 1.5e-2 |
| NOD-like receptor signaling pathway | RELA, IL6, MAPK1, MAPK3 | 1.5e-2 |
| Toll-like receptor signaling pathway | RELA, IL6, MAPK1, MAPK3, PIK3CA | 1.8e-2 |
| Ras signaling pathway | MET, RELA, RAF1, FLT1, MAPK1, MAPK3, PIK3CA | 2.0e-2 |
| Adherens junction | MET, ERBB2, MAPK1, MAPK3 | 3.0e-2 |
| Chemokine signaling pathway | BRAF, RELA, RAF1, MAPK1, MAPK3, PIK3CA | 3.2e-2 |
| MAPK signaling pathway | BRAF, RELA, RAF1, HSPB1, MAPK1, MAPK3, TP53 | 3.4e-2 |
| cAMP signaling pathway | BRAF, RELA, RAF1, MAPK1, MAPK3, PIK3CA | 4.0e-2 |
| Signaling pathways regulating pluripotency of stem cells | HNF1A, RAF1, MAPK1, MAPK3, PIK3CA | 4.4e-2 |
| Fc gamma R-mediated phagocytosis | RAF1, MAPK1, MAPK3, PIK3CA | 4.6e-2 |
| NF-kappa B signaling pathway | RELA, CARD11, PIDD1, PTGS2 | 5.0e-2 |

**Supplementary Table 4.** Total list of proteins modulated by arsenicals in prostate cancer.

| AKT1 | APEX1 | CREBBP | PTEN | FGFR4 | ZBTB16 | RXRA |
| --- | --- | --- | --- | --- | --- | --- |
| DCAF6 | ARG2 | CSRP1 | PTHLH | FHIT | ZBTB7A | SELENOP |
| EZH2 | AURKA | CTNNB1 | RUNX1 | FOXP4 | AKR1C3 | SERPINF1 |
| GSTT1 | BAD | CX3CL1 | SELENOS | GALNT3 | AR | SHBG |
| HMOX1 | BAX | CYP1B1 | SIRT1 | GGT1 | ARHGEF5 | SSX2 |
| IL6 | CALCA | DDR1 | STMN1 | GOLGA4 | BRCA2 | SULT1A1 |
| INS | CAV1 | DNASE1L2 | TCEAL7 | GREB1 | CALR | SULT2A1 |
| MTAP | CLDN7 | EGF | TMEFF2 | HIP1 | CAPNS1 | TCN2 |
| MLH1 | CLIC4 | EPHX1 | TYMS | IL16 | CAV2 | TLR6 |
| NFE2L2 | CLPTM1L | ESR1 | VDR | IRAK4 | CBR1 | TMSB4X |
| NQO1 | IGFBP3 | ESR2 | VEGFA | IVNS1ABP | CBX1 | TPD52L1 |
| STAT3 | IL10 | FOLH1 | VIM | JAK2 | CHEK2 | TPP1 |
| ACSL4 | IL1RN | FOXA3 | XRCC1 | JUP | CLDN3 | TRAF1 |
| ADI1 | IL2 | GHR | ZFHX3 | KDELR1 | CTSB | TST |
| BAP1 | IRS1 | GPX3 | ABCC4 | MAD1L1 | CXCL12 | TXNDC5 |
| BCL2 | ITGB3 | GSK3B | ABCG5 | MBD2 | CYP19A1 | WNT4 |
| BMP7 | KEAP1 | GSTM1 | ABR | MTHFR | DAB2IP | ZFAND5 |
| BNIP3 | LPAR1 | GSTP1 | ACHE | MYH14 | EHBP1 | ZMYM3 |
| BRD4 | MAPK3 | HIF1A | ACRBP | NFIC | ERBB3 | GNG5 |
| CASP9 | MIF | HNF1B | ANXA3 | NPPA | ETV1 | IL24 |
| CCND1 | MMP9 | HNRNPH1 | ANXA4 | OLFM1 | ETV4 | MBTPS1 |
| CCND2 | MYC | HOXB13 | ATF3 | PDE4D | GSTK1 | AHCYL2 |
| CDH1 | MYCL | HOXD3 | ATM | PLAUR | HIST1H2BG | CYP3A43 |
| CDH13 | NOS3 | HSP90B1 | ATR | PODXL | HSD17B1 | HSD3B2 |
| CDKN1A | PAK6 | ID3 | B2M | PON1 | HSD3B1 |  |
| CDKN1B | PARP1 | IGF1 | BMPR1B | PPARA | HSP90AB1 |  |
| CLU | PIK3CD | IGFBP6 | BRAF | PPFIBP2 | IGFBP7 |  |
| CRYAB | PLAU | IL18 | BRCA1 | PRDX2 | LAMC1 |  |
| CXCL8 | PTGS2 | ITPR1 | CCHCR1 | PRKCZ | LDHB |  |
| CYP17A1 | SERPINA3 | KLF6 | CD9 | PRSS8 | LPL |  |
| CYP1A1 | SERPINE1 | LAMB2 | CPNE3 | RNASEL | MAP3K7 |  |
| CYP3A4 | SLC39A1 | LEP | CREG1 | RNF130 | MME |  |
| EGFR | SOD2 | LIFR | CRYL1 | RRAS | MMP13 |  |
| EHF | STARD10 | MDM2 | CTBP2 | SERINC3 | MSMB |  |
| EMP1 | TERT | MPO | CTSD | SIL1 | MXI1 |  |
| ERBB2 | TGFA | MT2A | CYP2E1 | SPINK1 | NAT1 |  |
| ERCC2 | TGFB1 | NPR3 | CYP7B1 | SULT2B1 | NCOA2 |  |
| FBLN1 | TGFBR2 | NR3C1 | DEFB1 | TERC | NDRG1 |  |
| FGF2 | TLR4 | NRP1 | DNMT1 | TET2 | PAWR |  |
| GADD45A | TNFRSF21 | OGG1 | DNMT3B | TJP3 | PDZK1 |  |
| GDF15 | TP53 | P4HB | EGR1 | TNFSF10 | PGAM2 |  |
| GJA1 | ZFP36L2 | PCDH8 | ELAC2 | TOP2A | PGRMC1 |  |
| GSTA1 | ADRB2 | PDIA3 | EPCAM | TPBG | PHGDH |  |
| GSTM3 | AGR2 | PENK | ERP29 | TUSC3 | PIK3CA |  |
| GSTO1 | AHR | PKP3 | EZR | UMPS | PRNP |  |
| HDAC6 | ALAD | PLEK2 | FAF2 | USO1 | PTPRK |  |
| HSPA1A | ALDH1A2 | PRKDC | FASLG | VAV3 | RASD1 |  |
| ICAM1 | ANXA1 | PSCA | FGF10 | VPS52 | REC8 |  |

**Supplementary Table 5.** Molecular pathways in which the proteins are involved in prostate cancer with the relative p-value scores (p values < 0.05 are considered as significant).

| **PATHWAY** | **GENES** | **p-value** |
| --- | --- | --- |
| Pathways in cancer | AKT1, BRAF, BAX, BAD, BCL2  BRCA2, CXCL12, CXCL8, CTBP2, CREBBP, FASLG, GNG5, MDM2, TRAF1  WNT4, AR, CDH1, CASP9, CTNNB1, CCND1, CDKN1A, CDKN1B, EGFR, EGF, ERBB2, FGF10, FGF2, GSK3B, HSP90AB1, HSP90B1, HIF1A, IGF1, IL6, JUP, LAMB2, LAMC1, LPAR1, MMP9, MAPK3, MLH1, PTEN, PIK3CA, PIK3CD, PTGS2, RXRA, RUNX1, STAT3, TGFA, TGFB1, TGFBR2, TP53, MYC, VEGFA, ZBTB16 | 3.2e-20 |
| FoxO signaling pathway | AKT1, ATM, BRAF, BNIP3, CREBBP, FASLG, MDM2, CCND1, CCND2, CDKN1A, CDKN1B, EGFR, EGF, GADD45A, IGF1, IRS1, INS, IL10, IL6, MAPK3, PTEN, PIK3CA, PIK3CD, STAT3, SIRT1, SOD2, TGFB1, TGFBR2, TNFSF10 | 8.6e-16 |
| Proteoglycans in cancer | AKT1, BRAF, FASLG, MDM2, WNT4 CTNNB1, CAV1, CAV2, CCND1, CDKN1A, EGFR, ERBB2, ERBB3, ESR1, EZR, FGF2, HIF1A, ITPR1, IGF1, ITGB3, MMP9, MAPK3, PIK3CA, PIK3CD, PLAUR, PLAU, RRAS, STAT3, TLR4, TGFB1, TP53, MYC, VEGFA | 2.0e-14 |
| PI3K-Akt signaling pathway | AKT1, BAD, BCL2, BRCA1, FASLG, GNG5, JAK2, MDM2, CASP9, CCND1, CCND2, CDKN1A, CDKN1B, EGFR, EGF, FGF10, FGF2, FGFR4, GSK3B, GHR, HSP90AB1, HSP90B1,IGF1, IRS1, INS, ITGB3, IL2, IL6, LAMB2, LAMC1, LPAR1, MAPK3, NOS3, PTEN, PIK3CA, PIK3CD, RXRA, TLR4, TP53, MYC, VEGFA | 5.5e-13 |
| HIF-1 signaling pathway | AKT1, BCL2, CREBBP, CDKN1A, CDKN1B, EGFR, EGF, ERBB2, HIF1A, INS, IL6, MAPK3, NPPA, PIK3CA, PK3CD, SERPINE1, STAT3, TLR4, VEGFA | 2.2e-11 |
| Chemical carcinogenesis | NAT1, CYP1A1, CYP1B1, CYP2E1, CYP3A4, CYP3A43, EPHX1, GSTA1, GSTK1, GSTM1, GSTM3, GSTO1, GSTP1, GSTT1, PTGS2, SULT1A1, SULT2A1 | 3.8e-10 |
| Hepatitis B | AKT1, BAX, BAD, BCL2, CXCL8, CREBBP, FASLG, CASP9, CCND1, CDKN1A, CDKN1B, IL6, MMP9, MAPK3, PTEN, PIK3CA, PIK3CD, STAT3, TLR4, TGFB1, TP53, MYC | 5.3e-9 |
| MicroRNAs in cancer | ATM, BCL2, BRCA1, CREBBP, DNMT1, DNMT3B, MDM2, CCND1, CCND2, CDKN1A, CDKN1B, CYP1B1, EZH2, EGFR, ERBB2, ERBB3, EZR, IRS1, ITGB3, MMP9, PTEN, PLAU, PTGS2, STAT3, SIRT1, STMN1, TP53, MYC, VEGFA, VIM | 2.5e-8 |
| Transcriptional misregulation in cancer | ATM, CXCL8, ETV1, ETV4, MDM2, SSX2, TRAF1, CCND2, CDKN1A, CDKN1B, IGF1, IGFBP3, IL6, JUP, MMP9, MPO, PLAU, RXRA, RUNX1, TGFBR2, TP53, MYC, ZBTB16 | 1.5e-8 |
| Thyroid hormone signaling pathway | AKT1, BAD, CREBBP, MDM2, WNT4, CASP9, CTNNB1, CCND1, ESR1, GSK3B, HIF1A, ITGB3, MAPK3, NCOA2, PIK3CA, PIK3CD, RXRA, TP53, MYC | 1.7e-8 |
| p53 signaling pathway | ATM, ATR, BAX, MDM2, CASP9, CHEK2, CCND1, CCND2, CDKN1A, GADD45A, IGF1, IGFBP3, PTEN, SERPINE1, TP53 | 1.8e-8 |
| ErbB signaling pathway | AKT1, BRAF, BAD, CDKN1A, CDKN1B, EGFR, EGF, ERBB2, ERBB3, GSK3B, MAPK3, PAK6, PIK3CA, PIK3CD, TGFA, MYC | 8.5e-8 |
| Focal adhesion | AKT1, BRAF, BAD, BCL2, CTNNB1, CAV1, CAV2, CCND1, CCND2, EGFR, EGF, ERBB2, GSK3B, IGF1, ITGB3, LAMB2, LAMC1, MAPK3, PAK6, PTEN, PIK3CA, PIK3CD, VEGFA, VAV3 | 1.5e-7 |
| Steroid hormone biosynthesis | AKR1C3, CYP1A1, CYP1B1, CYP17A1, CYP19A1, CYP2E1, CYP3A4, CYP7B1, HSD3B1, HSD17B1, SULT2B1 | 1.8e-6 |
| Prolactin signaling pathway | AKT1, JAK2, CCND1, CCND2, CYP17A1, ESR1, ESR2, GSK3B, INS, MAPK3, PIK3CA, PIK3CD, STAT3 | 2.2e-6 |
| Ovarian steroidogenesis | AKR1C3, CYP1A1, CYP1B1, CYP17A1, CYP19A1, HSD3B1, HSD3B2, HSD17B1, IGF1, INS, PTGS2 | 2.6e-6 |
| Cell cycle | ATM, ATR, CREBBP, MAD1L1, MDM2, CHEK2, CCND1, CCND2, CDKN1A, CDKN1B, GSK3B, GADD45A, PRKDC, TGFB1, TP53, MYC | 8.9e-6 |
| Signaling pathways regulating pluripotency of stem cells | AKT1, JAK2, WNT4, BMPR1B, CTNNB1, FGF2, FGFR4, GSK3B, ID3, IGF1, LIFR, MAPK3, PIK3CA, PIK3CD, STAT3, MYC, ZFHX3 | 9.3e-6 |
| Apoptosis | AKT1, ATM, BAX, BAD, BCL2, FASLG, CASP9, PIK3CA, PIK3CD, TNFSF10, TP53 | 2.4e-5 |
| Central carbon metabolism in cancer | AKT1, EGFR, ERBB2, HIF1A, MAPK3, PTEN, PIK3CA, PIK3CD, PGAM2, TP53, MYC | 3.2e-5 |
| Jak-STAT signaling pathway | AKT1, CREBBP, JAK2, CCND1, CCND2, GHR, IL10, IL2, IL24, IL6, LEP, LIFR, PIK3CA, PIK3CD, STAT3, MYC | 5.8e-5 |
| Estrogen signaling pathway | AKT1, EGFR, ESR1, ESR2, HSP90AB1, HSP90B1, HSPA1A, ITPR1, MMP9, MAPK3, NOS3, PIK3CA, PIK3CD | 7.0e-5 |
| Rap1 signaling pathway | AKT1, BRAF, CDH1, CTNNB1, EGFR, EGF, FGF10, FGF2, FGFR4, IGF1, INS, ITGB3, LPAR1, MAPK3, PIK3CA, PIK3CD, PRKCZ, RRAS, VEGFA | 1.3e-4 |
| Cytokine-cytokine receptor interaction | CXCL12, CXCL8, CX3CL1, FASLG, TNFRSF21, BMP7, BMPR1B, EGFR, EGF, GHR, IL10, IL18, IL2, IL6, LEP, LIFR, TGFB1, TGFBR2, TNFSF10, VEGFA | 1.4e-4 |
| Glutathione metabolism | GGT1, GSTA1, GSTK1, GSTM1, GSTM3, GSTO1, GSTP1, GSTT1, GPX3 | 1.9e-4 |
| Protein processing in endoplasmic reticulum | BAX, BCL2, SIL1, CALR, CRYAB, ERP29, HSP90AB1, HSP90B1, HSPA1A, MBTPS1, NFE2L2, P4HB, PDIA3, SELENOS, TXNDC5, TUSC3 | 3.3e-4 |
| NF-kappa B signaling pathway | ATM, BCL2, CXCL12, CXCL8, TRAF1, ICAM1, IRAK4, MAPK3, PLAU, PTGS2, TLR4 | 4.4e-4 |
| Neurotrophin signaling pathway | AKT1, BRAF, BAX, BAD, BCL2, FASLG, GSK3B, IRS1, IRAK4, MAPK3, PIK3CA, PIK3CD, TP53 | 4.4e-4 |
| mTOR signaling pathway | AKT1, BRAF, IGF1, IRS1, INS, MAPK3, PTEN, PIK3CA, PIK3CD | 4.8e-4 |
| Insulin resistance | AKT1, GSK3B, IRS1, INS, IL6, NOS3, PPARA, PTEN, PIK3CA, PIK3CD, PRKCZ, STAT3 | 6.5e-4 |
| VEGF signaling pathway | AKT1, BAD, CASP9, MAPK3, NOS3, PIK3CA, PIK3CD, PTGS2, VEGFA | 6.8e-4 |
| Viral carcinogenesis | BAX, BAD, CREBBP, MAD1L1, MDM2, TRAF1, CCND1, CCND2, CDKN1A, CDKN1B, HIST1H2BG, HDAC6, MAPK3, PIK3CA, PIK3CD, STAT3, TP53 | 8.7e-4 |
| Cysteine and methionine metabolism | DNMT1, DNMT3B, ADI1, AHCYL2, LDHB, MTAP, TST | 1.2e-3 |
| Drug metabolism - cytochrome P450 | CYP2E1, CYP3A4, GSTA1, GSTK1, GSTM1, GSTM3, GSTO1, GSTP1, GSTT1 | 1.4e-3 |
| TNF signaling pathway | AKT1, CX3CL1, TRAF1, ICAM1, IL6, MMP9, MAPK3, MAP3K7, PIK3CA, PIK3CD, PTGS2 | 2.1e-3 |
| Ras signaling pathway | AKT1, BAD, FASLG, GNG5, EGFR, EGF, FGF10, FGF2, FGFR4, IGF1, INS, MAPK3, PAK6, PIK3CA, PIK3CD, RRAS, VEGFA | 2.4e-3 |
| Regulation of actin cytoskeleton | BRAF, EGFR, EGF, EZR, FGF10, FGF2, FGFR4, INS, ITGB3, MAPK3, PAK6, PIK3CA, PIK3CD, RRAS, TMSB4X, VAV3 | 3.1e-3 |
| Hippo signaling pathway | WNT4, BMP7, BMPR1B, CDH1, CTNNB1, CCND1, CCND2, GSK3B, PRKCZ, SERPINE1, TGFB1, TGFBR2, MYC | 3.3e-3 |
| MAPK signaling pathway | AKT1, BRAF, FASLG, EGFR, EGF, FGF10, FGF2, FGFR4, GADD45A, HSPA1A, MAPK3, MAP3K7, RRAS, STMN1, TGFB1, TGFBR2, TP53, MYC | 3.3e-3 |
| Sphingolipid signaling pathway | AKT1, BAX, BCL2, CTSD, MAPK3, NOS3, PTEN, PIK3CA, PIK3CD, PRKCZ, TP53 | 5.1e-3 |
| T cell receptor signaling pathway | AKT1, GSK3B, IL10, IL2, MAPK3, MP3K7, PAK6, PIK3CA, PIK3CD, VAV3 | 5.7e-3 |
| Progesterone-mediated oocyte maturation | AKT1, BRAF, MADL1L1, HSP90AB1, IGF1, INS, MAPK3, PIK3CA, PIK3CD | 6.6e-3 |
| Chemokine signaling pathway | AKT1, BRAF, CXCL12, CXCL8, CX3CL1, GNG5, JAK2, GSK3B, MAPK3, PIK3CA, PIK3CD, PRKCZ, STAT3, VAV3 | 6.7e-3 |
| Toll-like receptor signaling pathway | AKT1, CXCL8, IRAK4, IL6, MAPK3, MAP3K7, PIK3CA, PIK3CD, TLR4, TLR6 | 6.8e-3 |
| Adipocytokine signaling pathway | AKT1, JAK2, ACSL4, IRS1, LEP, PPARA, RXRA, STAT3 | 6.9e-3 |
| Adherens junction | CREBBP, CDH1, CTNNB1, EGFR, ERBB2, MAPK3, MAP3K7, TGFBR2 | 7.5e-3 |
| Aldosterone-regulated sodium reabsorption | IGF1, IRS1, INS, MAPK3, PIK3CA, PIK3CD | 7.7e-3 |
| NOD-like receptor signaling pathway | CXCL8, HSP90AB1, HSP90B1, IL18, IL6, MAPK3, MAP3K7 | 8.0e-3 |
| Regulation of lipolysis in adipocytes | AKT1, ADRB2, IRS1, INS, PIK3CA, PIK3CD, PTGS2 | 8.7e-3 |
| Leukocyte transendothelial migration | CXCL12, CTNNB1, CLDN3, CLDN7, EZR, ICAM1, MMP9, PIK3CA, PIK3CD, VAV3 | 1.3e-2 |
| AMPK signaling pathway | AKT1, CCND1, IGF1, IRS1, INS, LEP, MAP3K7, PIK3CA, PIK3CD, SIRT1 | 1.6e-2 |
| TGF-beta signaling pathway | CREBBP, BMP7, BMPR1B, ID3, MAPK3, TGFB1, TGFBR2, MYC | 1.8e-2 |
| cAMP signaling pathway | AKT1, ABCC4, BRAF, BAD, CREBBP, ADRB2, MAPK3, PPARA, PIK3CA, PIK3CD, PDE4D, RRAS, VAV3 | 2.5e-2 |
| Wnt signaling pathway | CTBP2, CREBBP, WNT4, CTNNB1, CCND1, CCND2, GSK3B, MAP3K7, TP53, MYC | 3.3e-2 |
| Insulin signaling pathway | AKT1, BRAF, BAD, GSK3B, IRS1, INS, MAPK3, PIK3CA, PIK3CD, PRKCZ | 3.3e-2 |
| Arachidonic acid metabolism | AKR1C3, CBR1, CYP2E1, GGT1, GPX3, PTGS2 | 4.8e-2 |

**Supplementary Table 6.** Mutational status of all the 327 nodes in linking region between three cancer networks.

| Gene | BlC | KiC | PrC |
| --- | --- | --- | --- |
| SIRT1 | x | x | x |
| NFYB |  |  |  |
| NFYA | x |  |  |
| VCP | x | x | x |
| DAXX | x | x | x |
| SKP1 |  |  |  |
| KDR | x | x | x |
| PARK7 |  |  | x |
| ATF3 |  |  | x |
| CDKN2A | x | x |  |
| MAPK11 | x |  |  |
| PIN1 |  |  |  |
| SCAMP1 |  |  |  |
| HSPB1 |  |  |  |
| PIAS4 | x |  | x |
| UBE3A | x | x | x |
| BMP1 |  | x | x |
| STAT1 | x | x | x |
| PHC3 |  |  | x |
| PPP1R13L | x | x | x |
| AATF | x |  |  |
| MAPK1 |  |  | x |
| BAX |  |  |  |
| BCR |  |  | x |
| PIAS2 |  | x |  |
| HACD3 |  |  | x |
| LAMA4 | x | x | x |
| PPP2R1A | x |  |  |
| VRK1 | x |  |  |
| ISL1 | x | x |  |
| FOXP3 |  |  |  |
| PSMC5 |  |  | x |
| KDM4A | x | x | x |
| PBK | x |  |  |
| HSPD1 | x | x | x |
| PPARGC1A | x | x | x |
| RBBP5 | x | x | x |
| PSMC4 |  |  |  |
| ANXA2 | x |  | x |
| WDR82 | x |  | x |
| HSPA9 | x | x | x |
| RXRA | x |  | x |
| SETD1A | x | x | x |
| ASH2L | x | x | x |
| PKM | x |  |  |
| S100A4 |  |  |  |
| CHMP2B |  | x |  |
| MYC | x |  | x |
| PLP2 | x |  |  |
| MDM4 |  |  | x |
| YY1 |  |  |  |
| NFKB1 | x | x | x |
| BCL2 |  |  |  |
| BCL2L1 | x |  |  |
| SREBF1 | x | x | x |
| CSNK1D |  |  |  |
| PPP2R5C |  |  |  |
| DDX5 | x |  |  |
| CREBBP | x | x | x |
| HSPA1B |  |  |  |
| RPGRIP1L | x | x | x |
| CREB1 |  |  | x |
| USF2 | x | x |  |
| MAPK10 | x | x |  |
| KIF5B |  | x | x |
| HSP90AB1 | x | x | x |
| MEN1 | x | x | x |
| RPA1 | x | x | x |
| BRD7 | x | x |  |
| PSMC6 | x | x | x |
| CEP120 | x | x | x |
| CDC42 |  | x |  |
| PDCD4 | x | x |  |
| APPL1 | x | x | x |
| RNF20 |  | x | x |
| PSMB5 | x | x | x |
| MAP3K7 | x |  | x |
| USP11 |  | x |  |
| NR3C1 | x |  |  |
| DVL2 | x |  | x |
| USP39 | x | x |  |
| PSMA2 |  |  | x |
| EAF2 |  | x |  |
| COPS5 |  | x |  |
| USP7 | x | x | x |
| NEIL3 | x |  |  |
| HDAC1 |  | x | x |
| SAT1 |  | x |  |
| TCF4 |  |  | x |
| PTGES3 |  |  |  |
| STK4 | x |  | x |
| STAT3 |  | x | x |
| CBLC |  | x | x |
| GUSBP1 |  |  |  |
| UBE2N | x |  |  |
| CRK |  |  | x |
| CUL9 | x | x | x |
| YWHAG |  |  |  |
| TK1 | x | x |  |
| MPHOSPH6 | x | x |  |
| SMARCA4 | x | x | x |
| PRKAB1 |  |  |  |
| SSB | x |  | x |
| MALT1 | x | x |  |
| ARRB2 |  | x |  |
| RPS7 | x |  |  |
| UBE2V1 |  |  | x |
| CCL18 |  |  |  |
| RHPN2 | x | x | x |
| HDAC2 | x |  | x |
| BCL6 |  | x | x |
| PML | x | x | x |
| SNRPN |  | x | x |
| TUBA1C | x |  |  |
| AIPL1 | x |  |  |
| GPX2 |  |  | x |
| UBE2I | x |  | x |
| RPS3 |  | x |  |
| FCAMR |  |  | x |
| RCHY1 |  | x | x |
| HIF1AN | x |  |  |
| ZIC3 | x |  | x |
| YY2 |  |  |  |
| ERH |  |  |  |
| TOP1MT | x |  | x |
| OTUB1 |  |  |  |
| PADI1 | x |  | x |
| NABP1 |  | x |  |
| MAGEB18 | x |  |  |
| ARIH2 | x |  | x |
| SETD7 |  | x | x |
| IL4 |  | x | x |
| TP53BP1 | x | x | x |
| SEC22B |  |  |  |
| CUL2 | x | x | x |
| ELOC |  | x |  |
| GRB2 |  | x | x |
| CSNK2A1 | x |  | x |
| PARP1 | x | x | x |
| GSPT1 | x | x |  |
| IKBKE | x | x | x |
| CEP128 |  | x | x |
| APOH |  | x |  |
| CSE1L | x | x | x |
| VPS35 | x | x | x |
| WDR5 |  | x |  |
| RAB4A |  | x |  |
| ATR | x | x | x |
| UPF1 | x | x | x |
| FXYD6 |  |  |  |
| PHB |  | x |  |
| PIK3R1 | x | x | x |
| XRCC1 | x | x |  |
| MAPK13 | x | x | x |
| EIF4A2 | x | x |  |
| APTX | x |  |  |
| OTUD5 | x |  |  |
| PSMB3 |  |  |  |
| TRAF2 | x | x | x |
| ZNF385A | x |  |  |
| SHC1 |  | x | x |
| KAT2B | x | x | x |
| FBXO11 | x | x |  |
| ESR1 |  |  | x |
| ANK2 | x | x | x |
| VCAM1 | x | x | x |
| CCNE1 |  | x |  |
| NCOA2 | x | x | x |
| HMGB1 | x |  |  |
| CITED1 |  |  |  |
| IL4R | x | x | x |
| CDKN1A | x | x | x |
| PSMD11 | x | x | x |
| MDM2 |  |  | x |
| VDR | x | x | x |
| EP300 | x | x | x |
| CDK2 | x | x |  |
| MAP1LC3A | x |  |  |
| GABARAPL2 |  |  |  |
| GABARAPL1 |  |  |  |
| ACP1 |  | x | x |
| FOXO3 |  | x | x |
| ZCCHC10 |  |  | x |
| PHF1 | x |  | x |
| KAT5 | x |  | x |
| BRCA1 | x | x | x |
| SREBF2 | x | x | x |
| NDRG1 | x | x | x |
| CDK1 |  |  |  |
| STAT5A |  |  |  |
| FN1 | x | x | x |
| CCT5 | x |  |  |
| TP63 | x | x | x |
| STX1A |  | x | x |
| PABPC1 |  | x | x |
| DIABLO |  |  |  |
| RB1 | x | x | x |
| IKBKB | x |  | x |
| RAB7A |  | x |  |
| RIPK2 | x | x | x |
| CHUK | x | x | x |
| TRAF6 | x | x | x |
| TNFRSF1A |  | x | x |
| TRAF1 |  |  | x |
| NPM1 |  |  |  |
| BATF2 |  |  |  |
| EGFR | x | x | x |
| MYCBP |  | x |  |
| CALCOCO2 | x | x | x |
| LRRK2 | x | x | x |
| EGLN3 |  |  |  |
| AARSD1 |  |  |  |
| SUMO1 |  |  |  |
| TNFRSF1B | x |  | x |
| CASP9 | x | x | x |
| TARS |  | x | x |
| SSX2IP | x |  | x |
| RPS9 | x | x | x |
| PSMD2 |  | x | x |
| S100A9 | x |  |  |
| TMCO1 | x | x |  |
| AP2M1 | x |  |  |
| CCDC59 |  | x |  |
| RPS16 |  |  |  |
| SMAD3 | x | x | x |
| CEP135 | x | x | x |
| ICK | x |  | x |
| HIPK3 | x | x | x |
| PSMC2 | x | x | x |
| CEBPB | x |  | x |
| CLK3 |  | x | x |
| RB1CC1 | x | x | x |
| PARD6B | x | x |  |
| CALM3 |  |  |  |
| MAPKAPK5 |  |  | x |
| TWIST2 |  |  |  |
| PRKAB2 | x |  |  |
| NFKB2 | x | x |  |
| PPP1CC |  |  |  |
| TWIST1 |  |  |  |
| CEP152 | x | x | x |
| NINL | x | x | x |
| PRKCD | x | x |  |
| NIN | x | x | x |
| ARRB1 | x |  | x |
| CSNK1E | x |  | x |
| BCR/ABL fusion | excluded | excluded | excluded |
| CCDC155 |  | x | x |
| GFI1 |  |  |  |
| TP53 | x | x | x |
| PPIF |  |  | x |
| KIT | x |  | x |
| RELA | x | x | x |
| MET | x | x | x |
| SQSTM1 | x |  |  |
| UBB | x |  | x |
| PSMC3 | x |  |  |
| HSP90AA1 | x | x | x |
| JMY |  | x |  |
| PTK2 | x | x | x |
| KIAA0087 |  |  |  |
| PIAS1 |  | x |  |
| OFD1 | x | x | x |
| KMT2A | x | x | x |
| S100A8 | x |  | x |
| GRPEL1 | x |  |  |
| ZBTB16 | x | x | x |
| FAM173A |  |  | x |
| TP53BP2 | x | x | x |
| CCDC106 |  |  |  |
| WDR33 | x | x | x |
| MAPK8IP1 | x | x |  |
| RNF40 | x | x | x |
| TP53TG1 |  |  |  |
| STX5 | x |  |  |
| PNP |  | x |  |
| PLCG2 | x | x | x |
| NLK | x |  | x |
| GSTM4 |  |  |  |
| TJP2 | x | x | x |
| CXXC1 | x | x | x |
| DLEU1 |  |  |  |
| COX17 |  |  |  |
| PFDN5 |  | x | x |
| HSPA1L | x | x | x |
| EIF3E |  | x |  |
| ARL3 | x |  |  |
| FASN | x | x | x |
| ANXA3 |  | x |  |
| TMSB4X |  |  |  |
| SULT1E1 |  |  | x |
| PRMT2 |  |  |  |
| SERPINB9 | x |  | x |
| SMARCC1 | x | x | x |
| PCDHA4 | x |  | x |
| TBP |  | x |  |
| PAFAH1B3 |  |  |  |
| MAD2L1BP | x |  |  |
| ITSN1 | x | x | x |
| EIF2S2 |  | x |  |
| ATXN2 | x | x | x |
| GNL3 | x |  |  |
| SPICE1 | x | x | x |
| TTN | x | x | x |
| WDR48 | x |  | x |
| BTBD2 | x |  | x |
| CDKN2C |  | x |  |
| CTNNB1 | x | x | x |
| FLT1 | x | x | x |
| BMX | x | x |  |
| HIPK1 | x | x | x |
| MAP2K7 | x | x | x |
| SMAD2 | x | x | x |
| SHANK2 | x | x | x |
| WRN | x | x |  |
| THAP8 | x |  |  |
| MAPK8 | x |  |  |
| UBA1 | x | x | x |
| CUL7 | x | x | x |
| SIN3A | x | x | x |
| CABLES1 | x | x | x |
| CNTF | x |  | x |
| RAD51 | x | x | x |
| ZNF24 | x | x | x |
| PPA1 | x |  |  |
| PRRC2C | x | x | x |

**Supplementary Table 7.** Kaplan-Meyer survival analysis has been applied to examine the association between HUBs that link three cancer networks and poor patient survival by PROGgeneV2 tool. We report the genes that have shown a statistically significant correlation to poor patient survival with the related p-value and information about the database used for the analysis. Moreover, we evidence in red or in green the p-values related to the genes for which the high or down expression is associated to poor patient survival.

| **Node** | **p-value** | **cancer** | **Database** |
| --- | --- | --- | --- |
| **MAP3K7** | 0.038 | bladder | TCGA_BLCA |
| **NR3C1** | 0.024 | kidney | TCGA_KIRC |
| **PABPC1** | 0.034 | bladder | TCGA_BLCA |
|  | 0.00031 | kidney | TCGA_KIRC |
| **NDRG1** | 0.0061 | bladder | TCGA_BLCA |
|  | 0.0000085 | prostate | GSE16560 |
| **RELA** | 0.000384 | kidney | TCGA_KIRC |
|  | 0.0035 | prostate | GSE16560 |

**Supplementary Figure 1. Alteration analysis of genes that codify for the proteins modulated by arsenicals in KiC and PrC.** The analysis was performed on TCGA_KIRC and TCGA_PRAD datasets using cBioPortal tool. The meaning of the colors associated to alterations is reported in the legend.

**Supplementary Figure 2. Evaluation of topological properties of KiC network**. (A) node degree distribution, (B) average clustering coefficient, (C) stress centrality, (D) closeness cenytrality and (E) betweenness centrality measure.

**Supplementary Figure 3. STRING analysis on proteins modulated by KiC.** Network obtained for ninety-six proteins modulated by arsenicals considering a confidence score value of 0.4 (A) and of 0.7 (B). Nodes are shown as circle and interaction as line. In detail, blue water and fucsia lines refers to known interactions; green, red and blue lines display predicted interactions while yellow, black and pink lines are associated to others types of interactions including textmining, co-expression and protein homology, respectively.

**Supplementary Figure 4. Evaluation of topological properties of PrC network.** (A) node degree distribution, (B) average clustering coefficient, (C) stress centrality, (D) closeness centrality and (E) betweenness centrality measure for prostate cancer.

**Supplementary Figure 5. STRING analysis on proteins modulated by PrC.** Network obtained for three hundred and thirteen proteins modulated by arsenicals considering a confidence score value of 0.4 (A) and of 0.7 (B). Nodes are shown as circle and interaction as line. In detail, blue water and fucsia lines refers to known interactions; green, red and blue lines display predicted interactions while yellow, black and pink lines are associated to others types of interactions including textmining, co-expression and protein homology, respectively.

**Supplementary Figure 6. Evaluation of topological properties of merged network**. (A) node degree distribution, (B) average clustering coefficient, (C) stress centrality, (D) closeness cenytrality and (E) betweenness centrality measures.
